# The Effects of the Flint water crisis on the educational outcomes of school-age children

**DOI:** 10.1126/sciadv.adk4737

**Published:** 2024-03-13

**Authors:** Sam Trejo, Gloria Yeomans-Maldonado, Brian Jacob

**Affiliations:** ^1^ Department of Sociology, Princeton University, Princeton, NJ 08544, USA.; ^2^ Office of Population Research, Princeton University, Princeton, NJ 08544, USA.; ^3^ Ford School of Public Policy, University of Michigan, Ann Arbor, MI 48109, USA.; ^4^ National Bureau of Economic Research, Cambridge, MA 02138, USA.

## Abstract

In 2014, the municipal water source in Flint, Michigan was switched, causing lead from aging pipes to leach into the city’s drinking water. While lead exposure in Flint children increased modestly on average, some children were exposed to high lead levels. Surveys of Flint residents show the water crisis was also associated with increased levels of stress, anxiety, and depression. We use Michigan’s administrative education data and utilize synthetic control methods to examine the impact of the crisis on Flint’s school-age children. We find decreases in math achievement and increases in special needs classification, even among children living in homes with copper (rather than lead) water service lines. Low socioeconomic status students and younger students experienced the largest effects on math achievement, and boys experienced the largest effects on special needs classification. Our results point toward the broad negative effects of the crisis on children and suggest that existing estimates may substantially underestimate the overall societal cost of the crisis.

## Teaser

Students experienced decreases in math achievement and increases in special needs classification following the Flint water crisis.

## INTRODUCTION

In January 2016, the eyes of America became fixed firmly upon Flint, Michigan. National news outlets reported that Flint’s water supply had been contaminated with high levels of lead. Then-governor Rick Snyder declared a state of emergency and called in the National Guard to distribute bottled water. Within weeks, the Flint water crisis (FWC) was classified as a federal disaster and the Environmental Protection Agency (EPA) took over management of the town’s water supply. By then, the roughly 100,000 citizens of Flint had been exposed to polluted water for over a year and a half. The mostly Black, industrial city quickly became a national symbol for governmental negligence and racial injustice (*1*).

Previous research has found that while the FWC led to relatively modest increases in child blood lead levels on average (*2*), it resulted in substantial increases among certain children. Hanna-Attisha *et al.* (*3*) find that the percentage of Flint children with elevated blood lead levels doubled, from roughly 2.5 to 5%. Lead exposure in early childhood is associated with a host of negative outcomes, including increased anxiety (*4*), increased behavioral problems (*5*), decreased executive functioning (*6*), decreased academic achievement (*7*), decreased brain volume (*8*), higher rates of criminal offending (*9*), and decreased social mobility (*10*). Given that the consequences of lead contamination are known to be most severe for infants and toddlers, it is unclear if the lead exposure caused by the crisis would be expected to have had a detectable impact on educational outcomes in Flint in the short run. However, the crisis also had a profoundly negative psychosocial impact on Flint residents, who reported increased anxiety, depression, sleep problems, and worries about physical health (*11*).

To quantify the impact of the FWC on several important educational outcomes, we use the universe of longitudinal student-level education records for the State of Michigan matched to address-level information on water service line material in Flint. Public education records may well be the only longitudinal data source containing virtually all children living in Flint. We use a between-district analysis using synthetic control methods (SCMs) (*12**–**14*) to compare educational outcomes in Flint to outcomes in observationally similar school districts throughout Michigan. This analysis captures both the direct health impact of lead exposure and any broad effects resulting from the upheaval of the crisis on individual children.

The results of our synthetic control analysis suggest that the FWC induced a 0.14 SD decrease in math achievement and an 8% increase in the number of students with a qualified special educational need, with limited or no evidence for effects on either reading achievement or daily attendance. Using a treatment effect decomposition, we find that the negative impact on math achievement was larger for students below (rather than above) the median of Flint’s socioeconomic distribution and for students in grades 3 to 5 (rather than grades 6 to 8). The observed effects on special needs classification, on the other hand, were driven largely by effects on male (rather than female) students.

To better understand these observed treatment effects, we use a unique dataset of home service line inspections collected shortly after the crisis. During the FWC, children living in homes with lead pipes consumed 4.5 times the amount of lead per day than children living in homes with copper pipes (*15*). We find little difference in the academic trajectories of Flint children living in homes with dangerous service lines (i.e., lead or galvanized steel) compared to children in in homes with safer service lines (i.e., copper). These results suggest that, for school-age children, the broad effects of the FWC were larger than the direct health effects of lead poisoning on the educational outcomes we consider. While our analysis does not generate positive evidence in support of specific mechanisms, the observed effects of the crisis writ large are larger than past studies would suggest that could result from lead exposure alone. This finding is consistent with the extant literature on the broad impacts of adverse community events. From a policy perspective, our findings suggest that existing estimates of the effects of lead exposure on child outcomes may substantially underestimate the overall cost of crises like Flint’s.

Our research contributes to the literature in several ways. First, we quantify the educational costs of a famous case of government mismanagement and document its broad effects on the outcomes of school-age children. Second, we explore the impact of lead on children who are exposed when they are above the age of five. Third, we highlight the indirect ways in which experiencing a disruptive community crisis can profoundly affect the educational development of school-age children.

The present study has important limitations. Foremost is that, because we observe only school-age children in our data, we cannot speak to the educational consequences of the FWC on infants or young children. However, Flint children who had yet to enter formal schooling were likely the most affected by increased lead exposure induced by the crisis, highlighting the importance of continued monitoring over time. Moreover, our analysis is limited to specific educational outcomes captured in state administrative data, such as standardized test scores and special needs classification. Hence, we are therefore unable to detect impacts on a variety of meaningful behaviors and skills. While our decomposition strategy using a child’s home service line helps us to separate the direct health effects of lead from the broad effects of the ensuing crisis, we cannot identify precise pathways through which any indirect effects may have operated. Finally, community organizations in Flint responded to the crisis by dramatically expanding the set of social, medical, and educational services available to children in Flint (for instance, the Flint Pediatric Public Health Initiative), suggesting that our estimates could understate the negative effects of the crisis alone.

### Background literature

#### The effects of lead poisoning

Lead is a powerful neurotoxin with no known safe level of exposure (*16*). While exposure has fallen notably over the past 40 years, more than 500,000 U.S. children under the age of five still have elevated blood lead levels (>5 μg/dl). Lead exposure is particularly dangerous for young children, who are comparatively small in size (and therefore more susceptible to low amounts of lead) and experiencing a critical period for brain development (*17*). Lead exposure during childhood is associated with a host of negative outcomes, including decreased cognitive ability, increased anxiety/depression, increased impulsivity, decreased brain volume, higher rates of criminal offending, and decreased social mobility (*18*). However, much of the existing research is correlational in nature and may suffer from confounding due to omitted variables, such as socioeconomic status (*19*). In addition, the extent to which lead exposure is associated with negative outcomes in children exposed after the age of five is less well understood.

A growing quasi-experimental literature documents the causal effects of lead on downstream health and human development. One approach leverages exogenous variation in exposure to lead resulting from public health programs that screen homes for exposed lead paint; Billings and Schnepel (*20*), Aizer *et al.* (*21*), and Sorensen *et al.* (*22*) use this empirical strategy and show a negative causal relationship between childhood exposure to lead and future math and reading test scores. While compelling, these studies using of blood lead measurements of children are not without their challenges. Measurement of lead is complicated by the fact that the amount of lead in the blood can fluctuate wildly, dissipates quickly after exposure, and is often not a reliable signal of the amount of lead in an individual’s body (*17*), producing attenuation bias.

Another quasi-experimental approach is to exploit large, regional changes in exposure to lead. For example, Reyes (2007; 2015) and Aizer and Currie (2017) show that the removal of lead from gasoline in the late 1970s explains part of the decrease of antisocial and risky behavior in adolescents and incarceration in adulthood; Grönqvist *et al.* (*23*) use Swedish data to show that lead exposure affects long-run outcomes via effects on both cognitive and socioemotional development. Historically, the expansion of lead pipes in the early 20th century corresponded with increases in infant mortality (*24*), decreases in military test scores (*25*), and increases in homicide rates (*26*).

Table S1 uses estimates from previous studies to benchmark the magnitude of the relationship between lead exposure and cognitive ability. Crucially, an increase of 0.5 μg/dl, the estimated change among children living in Flint during the crisis, would produce an expected 0.017 SD decrease in cognitive outcomes. These estimates come from studies that focus on the effects of lead exposure on younger children (see table S1). Because lead exposure typically peaks around age 2, as well as the autocorrelation of an individual’s lead exposure over time, the effect of lead on older children has not been well documented (*27*). We argue that estimates in childhood can serve as a conservative upper bound for the possible effects of lead in school-age children.

#### Lead contamination via drinking water

Lead has been a common material used in plumbing for over a thousand years. In 1900, 39 of the 46 largest cities in the United States used lead pipes (*28*). For lead to contaminate tap water, a system must both contain lead pipes and water corrosive enough to cause leaching. Lead in water is uniquely difficult to contain; while most contaminants can be filtered out at the treatment plant, lead typically enters drinking water at the end of the system through lead service lines, which run beneath the ground and connect individual residences to city water mains. In the United States today, 6.7 million homes, serving approximately 19 million Americans, are supplied by lead service lines. Even “lead-free” pipes can contain up to 8% lead, which has caused lead poisoning in cities that never had “lead” pipes (*29*). Finally, lead faucets and other fixtures are also sources of drinking water contamination.

#### The impacts of disruptive community crises

The neurotoxic effects of lead are not the only pathway through which the FWC may have negatively affected children. Social and psychological processes also have an important role to play in shaping the outcomes of individuals living in crisis-affected communities. For example, in assessing the long-term effects of the Chernobyl nuclear accident in Ukraine, a United Nations report concluded that the mental health impacts resulting from increased fear, anxiety, and trauma actually surpassed the physical health effects of exposure to radiation (*30*).

There are a range of mechanisms through which a community crisis may itself affect child development, which we collectively refer to as the “broad” effects of the FWC. We use the term to refer to the many ways in which the FWC may have impacted child development (beyond increasing the amount of lead in a child’s body). Traumatic events, such as terrorist attacks and natural disasters, are associated with negative psychological consequences for entire communities. Using interviews of individuals following major events such as the 1995 Oklahoma City bombing (*31*, *32*), the 11 September 2001 terrorist attacks (*33*, *34*), and Hurricane Katrina (*35*), researchers have documented persistent psychological distress and trauma in residents of the affected regions. This can even be true among those who were themselves not directly exposed to the crises (e.g., children who were not present for the events of the Oklahoma City bombing and lived miles away).

A more recent, causal literature leverages quasi-experimental variation in violent events across place and time to study the psychosocial effects of community-level trauma. Sharkey (*36*) and Rossin-Slater *et al.* (*37*) show that community exposure to deadly shootings negatively affects a child's academic performance and mental health, respectively. The effects on academic performance emerge almost immediately, and the effects on mental health persist for many years. Similarly, Gershenson and Tekin (*38*) show schools near the locations of the 2002 D.C. sniper attacks experienced decreases in academic achievement, with the overall effects being largely driven by the outsized impact on children in high poverty schools. A study of civil war and genocide in Cambodia (*39*) found that disruptions to primary education during civil conflicts decreased educational attainment and earnings decades later. In Mexico, increased regional exposure to violence resulting from the emergence of drug cartels led to negative impacts on the academic achievement and educational attainment of children (*40*).

In addition, racialized events within a community such as the FWC can produce a sense of social marginalization and subsequent civil unrest, which has been shown to affect academic performance. Gershenson and Hayes (*41*) show that the weeks of protests in Ferguson, Missouri, following the 2014 police killing of 18-year-old unarmed Black man Michael Brown caused decreases in math and reading achievement. These events may limit instructional time and learning by increasing absences, school closures, and disruption to daily routines. In addition, these out-of-school events may increase teacher or administrator turnover, which, in turn, affects students.

Last, there has been a concern that children in Flint will be labeled and/or stigmatized, similar to individuals with a psychiatric diagnosis (*42*) or a criminal record (*43*), as a result of the public perception of how the water crisis negatively affected children (*44*, *45*). This stigmatization may negatively affect development through processes such as interpersonal discrimination (*46*) and stereotype threat (*47*).

There is also reason to suspect that the FWC may not have affected all Flint students equally. Individuals and families with fewer economic, social, and cultural resources are often more limited in their ability to adapt to disruptive life events (*48*). Prior research has found that experiencing an earthquake in utero had a strong negative impact on the cognitive development of children from low socioeconomic status families but no effect among more advantaged families (*49*). Similarly, in utero radiation exposure has been shown to reduce academic achievement only among children from low socioeconomic status families (*50*). Last, the large average learning losses experienced during the COVID-19 pandemic were highly variable across communities, with the largest losses seen in high-minority and high-poverty districts (*51*).

### The FWC

#### Timeline

Since the 1960s, Flint residents had been supplied water from Lake Huron, provided by Detroit’s Water and Sewage Department. In 2011, with Flint’s government bankrupt, then-governor Rick Snyder appointed the first of a long string of emergency managers tasked with balancing the city’s budget. To reduce costs, the flow of water from Detroit was shut off and replaced by the Flint River, a small river that runs through its downtown. The Flint River’s water presented a challenge to treat due to high levels of bacteria and carbon concentrations (*52*) and because Flint’s own Water Service Center—a small facility traditionally maintained as a backup—was ill-prepared.

On 25 April 2014, Flint’s residents began receiving drinking water from the Flint River. A large fraction of Flint’s service lines were made of lead, causing the metal to begin leaching into the tap water (*53*). Almost immediately, residents began to complain about the color, taste, and odor of their water supply. While some Flint residents opted to begin consuming bottled water, many continued to drink the tap water as city and state officials insisted on its safety. Famously, the mayor of Flint appeared on local on local news to drink the tap water himself.

**Fig. 1.**
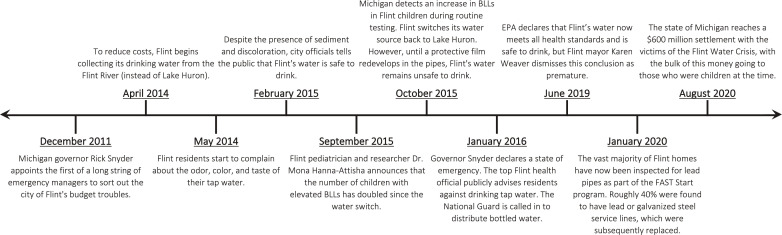
Timeline of the Flint water crisis.

In early 2015, local researchers and federal EPA employees raised concerns about elevated lead levels in the tap water of Flint residents and increased blood lead levels among Flint children. In October 2015, the city switched back to a water supply from Detroit, but Flint’s tap water remained unsafe to drink as a protective mineral film needed to develop over time inside the pipes. In January 2016, almost 18 months after the switch to water from the Flint River, then-Michigan Governor Rick Snyder declared a state of emergency and called in the National Guard to distribute bottled water. In August 2020, the State of Michigan reached a $600 million settlement with the victims of the crisis, with the bulk of the money going to those who were children at the time of the FWC. Today, the vast majority of lead service lines in Flint have been inspected and, if necessary, replaced. Despite the large amount of governmental and philanthropic investment in Flint over the past 6 years intended to help remediate the crisis, housing values have fallen over half a billion dollars, highlighting the lasting impact of the crisis on the town and its people (*54*). Figure 1 provides a timeline of the key events surrounding the FWC.

**Fig. 2.**
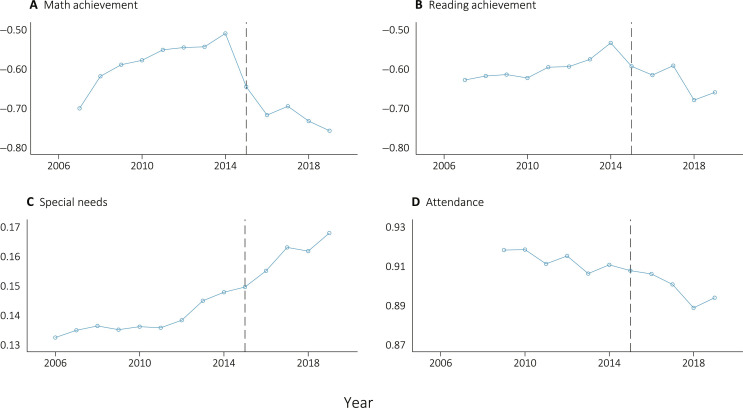
Mean educational outcomes over time in Flint. This figure displays descriptive trends in the mean academic outcomes for the Flint geographic district from 2006 to 2019. Data are taken from the Michigan Department of Education’s longitudinal administrative database. Black dotted lines represent the start of the posttreatment period (i.e., the first year of data after the FWC had begun). Math and reading achievement (**A** and **B**) are observed for only for grades 3 to 8 and are standardized within test subject, grade, and year to the overall state distribution scores. Math and reading achievement observations begin in 2007. Both special needs (**C**) and attendance (**D**) are observed in grades K-12, and special needs status observations begin in 2006, whereas attendance observations begin in 2009.

#### Prior research on the FWC

Prior research has found that blood lead levels in Flint children increased by roughly 0.5 μg/dl (0.2 SD) as a result of the crisis (*2*). In addition, the fraction of children identified with blood lead levels above 5 μg/dl roughly doubled, rising from 2.5 to 5% (*3*), with the greatest increases observed in the neighborhoods with the highest water lead levels. While any increase in lead exposure can be damaging, Zahran *et al.* (*2*) find that the average blood lead increase experienced by Flint children in prior research (an approximately 20% increase from the pretreatment period mean of 2.5 μg/dl) was relatively modest—similar to that of the typical seasonal change in lead exposure experienced by children in Flint from winter to summer (*2*, *55*). According to this research, even in the midst of the FWC, the fraction of child blood lead attributable to non-water sources (e.g., paint, soil dust, and airplane fuel) was larger than water lead sources (*15*). However, others in the Flint medical and public health community argue that the blood level measures are not reliable and likely understate the true extent of the exposure that children experienced. While the question of exactly how much increased lead exposure the FWC induced in Flint children is contested, we prefer the Zahran *et al.* (*2*) estimate of 0.5 blood lead levels (BLL) because of the study’s use of quasi-experimental methods. In addition, the authors test whether their estimates of BLL change may be driven by changes over time to the demographic and spatial characteristics of the children tested (these robustness checks find that key characteristics remained consistent throughout the FWC and the authors conclude that “the timing of exogenous shocks to population water-lead exposure risk appear independent of the demographic and spatial sampling protocols”).

There is mixed evidence on the causal effects of the FWC on neonatal outcomes. Grossman and Slusky (*56*) use difference-in-differences to compare childbirths in Flint to other Michigan cities before and after FWC and found a 12% decrease in fertility (which they attribute to preterm pregnancy loss) and that the overall health of Flint newborns decreased. Wang *et al.* (*57*) used a similar methodology and found that the frequency of low birth weight increased by more than 15%, with the greatest increase among Black newborns. However, Gómez *et al.* (*58*) found that lead levels in Flint women of childbearing age did not increase during the FWC and the subsequent 18-month time period, casting doubt on lead as a pathway for the observed effects.

Residents of Flint viewed the crisis as having a profoundly negative impact on their well-being. A recent review of 11 studies highlights the ways in which Flint residents perceived the crisis as increasing anxiety, depression, sleep problems, and worries about physical health (*11*). In addition, these studies found that residents reported coping with increases in risky health behaviors, such as smoking and alcohol misuse. Some studies also found that, among Flint residents, lower perceived tap water quality was associated with poorer mental and physical health and that the negative psychological consequences of the FWC continued even after the state of emergency was lifted. A recent causal study estimated a 11 percentage point increase in smoking and a 2% point decrease in breastfeeding among Flint mothers, resulting from stress related to the changes in water quality during the crisis (*59*).

Of course, the precise manifestation of the water contamination event in Flint as the “Flint water crisis,” as well as the stress, stigma, marginalization, and protest that it produced among local residents, was not inevitable. As with other pivotal racialized events, such as the widely publicized beating of Rodney King by police officers in 1991, the social crisis that emerged was the result of a bidirectional interaction between the events and their narrative understandings (*60*). Other studies explore how exactly the underlying public health crisis, combined with residents’ concerns, inaction by public officials, and the involvement of key researchers, produced what is now known as the FWC (*61*). In the present paper, we seek not to understand how the FWC came to be but instead to rigorously document its impact on the children of Flint.

## MATERIALS AND METHODS

### Data

Our primary data source is longitudinal student-level administrative educational records from the State of Michigan, provided by the Michigan Education Data Center. It consists of annual information on all students in Michigan public schools (including charter schools), from pre-kindergarten to high school, and contains demographics, enrollment information (including attendance and mobility), and outcomes such as academic achievement. These data also contain information from the U.S. Census and American Community Survey characterizing the demographic and socioeconomic characteristics of the neighborhoods in which students live.

We focus on four key educational outcomes: math achievement, reading achievement, special needs status, and daily attendance. Math and reading achievement are measured using annual state-administered educational assessments, which are given to students in grades 3 to 8 starting in 2007. We standardize these test scores at the grade-subject-year level using the distribution of all public school students in Michigan. Special needs status and daily attendance are observed for all students (i.e., K-12), with the former available since 2006 and the latter available starting in 2009. We choose these outcomes because (i) they are well-measured beginning many years before treatment and continuing through the FWC and (ii) they are good theoretical candidates to be affected by lead exposure and community crisis.

To construct a panel of Michigan districts, we must first assign each student to a school district. School choice is widespread in Michigan, and during the 2013–2014 school year, only 45% of public-school students living in Flint city limits attended Flint Community Schools, Flint’s zoned school district (the remaining 55% of students living in Flint attended charter schools or neighboring districts and are still captured in our data). Given that the FWC affected all children living in Flint regardless of where they attended school, we define our treatment group to include all students living within Flint, or identically, the geographic boundaries of Flint Community Schools. Similarly, our comparison districts are defined on the basis of students’ zoned school district (i.e., their geographic district) as opposed to the district they actually attend (i.e., their administrative district). For the remainder of the paper, we use the term district to refer to a geographic school district. To facilitate the SCM analysis, we collapse the student-level data into a geographic district-year panel from 2006 to 2019.

For use in our treatment effect decomposition analysis, we also create Flint district-year-subgroup panels using five dichotomous (or dichotomized) student-level characteristics: gender, grade, socioeconomic status, district mobility, and home service line material (lead or copper). With the exception of service line material, all of these measures are derived from Michigan’s administrative education records. A student’s grade (K-12) is dichotomized as ≤5 or ≥ 6. A student is considered “mobile” if they ever attend a district other than Flint or if they leave the Michigan public school system without graduating—otherwise, a student attends only Flint and is considered to be “immobile.”

A challenge for measuring socioeconomic heterogeneity in Flint is the coarseness of the economic disadvantage measure used by public schools: Nearly 90% of Flint students were categorized as economically disadvantaged each year. However, recent research using the Michigan administrative education data has shown that there is meaningful year-to-year variation in a student’s economically disadvantaged status and that those who qualify in a greater number of years are more disadvantaged than those who qualify in fewer years (*62*). To generate our Flint socioeconomic status variable, we restrict to a subsample of students who lived in Flint at least once from 2006 to 2019. We then separately average three dichotomous variables—economic disadvantage, receipt of Supplemental Nutrition Assistance Program (SNAP) benefits, and receipt of Temporary Assistance for Needy Families (TANF) benefits—across all years in which a student is observed. We then take the first principal component of these three averages and dichotomize it at the median. This principal component explains 49% of the overall variation and is correlated 0.64, 0.79, and 0.53 with average economic disadvantage, SNAP benefits, and TANF benefits, respectively.

Last, we constructed the service line material variable using data from inspections conducted by Flint’s FAST Start team. FAST Start was a program led by city and state officials that managed lead service line replacement following the FWC. The data were generously provided to us by academic researchers at the University of Michigan and the Georgia Institute of Technology who partnered with the FAST Start team to help with data management and refine the prediction of service line material (*63*). Using a probabilistic matching algorithm based on street number, street direction, street name, and street type, we were able to identify the service line material for 10,245 students, roughly 60% of students who were living in Flint and enrolled in Michigan public schools during the 2013–2014 school year. See the Supplementary Materials for more information on the service line data.

In Flint, lead service lines, galvanized steel service lines, and a small fraction of service lines made of nonstandard materials were all considered dangerous (and scheduled for replacement) because they could leach lead into the water supply. We henceforth use the term lead pipes to refer collectively to housing with any pipe material (lead, galvanized steel, or nonstandard materials) that increased the risk of lead contamination. In table S10, we show that our results are robust to alternative definitions. Only 2% of home that we include in our lead variable are composed only of other dangerous pipes (i.e., galvanized steel or nonstandard materials). The vast majority of service line inspections were conducted in 2018 and 2019, so most Flint families had no direct information about their service line material until near the end of our study window.

Past research used the same service line inspections data, combined with home water testing results, to show that service lines were a key source of lead exposure in Flint. During the peak of the crisis, children living in homes with lead service lines consumed 4.5 times the amount of lead per day than children residing in homes with copper pipes (*15*). The same study found that during the FWC, galvanized steel service lines, which we include in our primary lead treatment definition, were associated with 1.8 times more childhood lead consumption with copper pipes. In the Supplementary Materials, we confirm that similar patterns applied in our analytic sample; students in homes with lead or galvanized steel service lines were 4.7 percentage points (*P* < 0.01), or 77%, more likely to exhibit water lead levels above the EPA threshold of 15 parts per billion.

### Synthetic control analysis

We leverage a between-district analysis to determine the overall impact of the FWC on student outcomes. Using SCMs, we compare the academic trajectories of students living in Flint to those of students living in observationally similar districts using data spanning from 2006 to 2019.

#### Trends in educational outcomes in Flint

Figure 2 plots the trends of Flint’s academic outcomes over time. For math achievement, we observe a positive trend in the pretreatment period from 2007 to 2014 and then a drop of roughly 0.15 SDs in the first year following the water crisis. The negative trend that began in 2015 largely continues through 2019. The descriptive trend in reading achievement is similar but smaller in magnitude. With respect to special needs, we observe a positive trend before the crisis that appears to quicken following the water crisis. Turning to student attendance, we observe a steady decrease in attendance before the crisis that grows in 2017 and 2018. Figure S1 displays several additional Flint trends over time, including enrollment, the fraction of students tested, and the fraction of students living in Flint who attend Flint Community Schools.

The trends shown in Fig. 1 provide suggestive evidence that the FWC negatively affected student outcomes. However, it is possible that other factors may have played a role, ranging from changing economic conditions to changes in state education policies. For this reason, we turn to SCM to estimate how educational outcomes in Flint would have evolved in the absence of the water crisis.

#### Restricting district comparisons

In 2013–2014, Flint’s 16,210 zoned students in K-12 made it the ninth largest district in Michigan. Flint also has an exceptionally high fraction of economically disadvantaged students (89%) and fraction of Black students (76%), which complicate the identification of a valid comparison district elsewhere in Michigan. Using control districts too distant from Flint in covariate space might lead to an apples and orange comparisons. To ensure that we are comparing Flint to similar districts, we first exclude very small school districts (specifically, the 185 districts with fewer than 1000 students in the 2013–2014 school year) from our set of potential comparison districts. Relative to these remaining Michigan districts with at least 1000 students, Flint is at the 99th percentile in terms of fraction Black and fraction economically disadvantaged. Thus, we restrict our control sample to union of the set of districts in the top 10% of fraction Black and the set of districts in the top 10% of fraction economically disadvantaged. These criteria leave us with 54 potential control districts (see table S2 for the complete list).

**Fig. 3.**
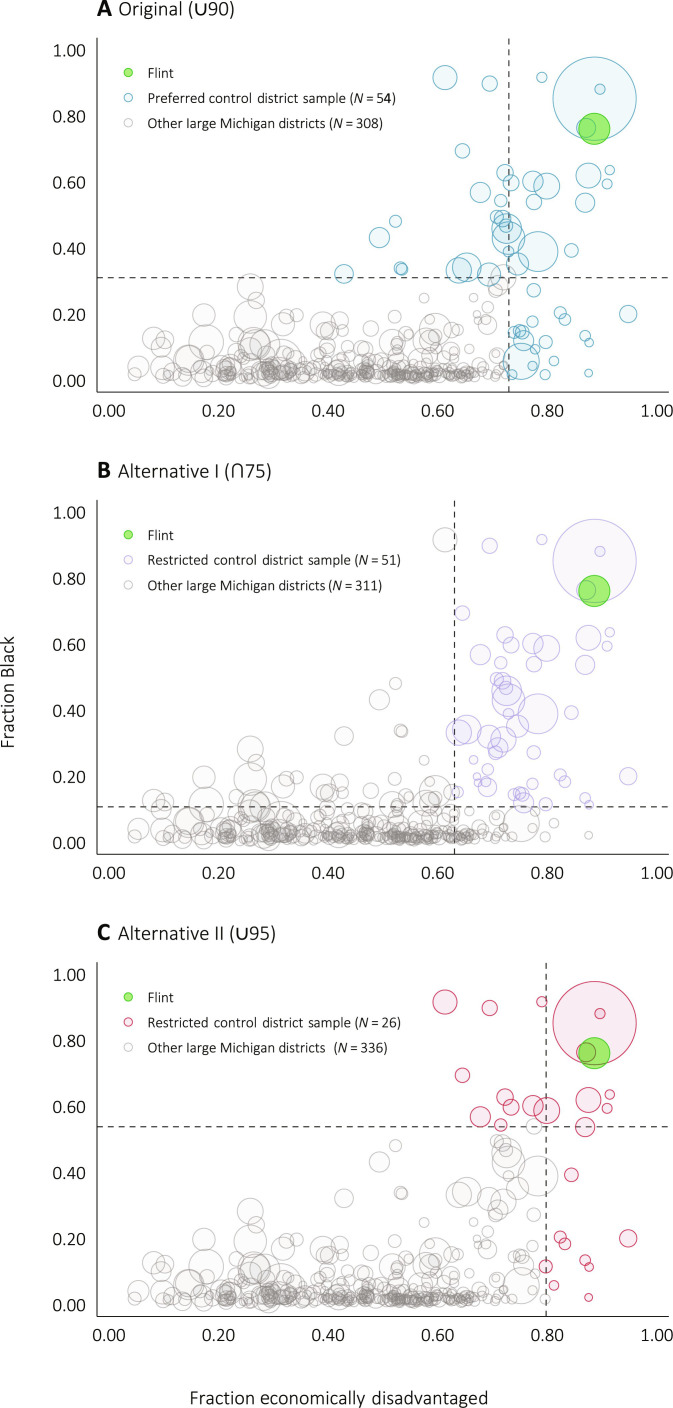
Synthetic control sample selection. Each circle represents a Michigan geographic school district. Data are taken from the Michigan Department of Education’s longitudinal administrative data base. All districts with enrollment greater than 1000 students are displayed, and the size of each school’s circle is proportional to its student enrollment. The highlighted districts represent the three different district samples used in our primary synthetic control model (**A**) and various robustness checks (**B** and **C**). In (A), dashed lines are at *X* = 0.73 and *Y* = 0.31, the 90th percentiles of fraction economically disadvantaged and fraction black, respectively. In (B), dashed lines are at *X* = 0.63 and *Y* = 0.11, the 75th percentiles. Last, in (C), dashed lines in (B) are at *X* = 0.8 and *Y* = 0.54, the 95th percentiles. All variables were measured in the 2013–2014 academic year. While 42 of the districts are the same across the ∪90 and the ∩75 districts samples, there are 23 districts that appear in one but not the other.

Figure 3A provides a graphical illustration of the sample selection process for our primary SCM analysis. Figure 3 (B and C) shows two alternative district sample restriction strategies, which we use as robustness checks: the intersection of the set of districts in the top 25% of fraction Black and the set of districts in the top 25% of fraction economically disadvantaged (∩75) as well as the union of the set of districts in the top 5% of fraction Black and the top 5% of fraction economically disadvantaged (∪95).

Table 1 displays descriptive statistics for all Michigan geographic districts for 2013–2014. While the 54 potential control districts more closely mirror Flint in terms of size and demographics, Flint has notably lower academic achievement than this group. Using our SCM algorithm will allow us to identify the weighted average of these 54 potential control districts that best approximates Flint’s outcome trends in the pretreatment period.

**Table 1.** Descriptive statistics on Michigan geographic districts, 2013–2014. Math and reading achievement are standardized within test subject, grade, and year to the overall state distribution scores. Math and reading achievement are observed for grades 3 to 8; all other variables are observed for grades K-12. This table contains geographic school district characteristics from the Michigan Department of Education’s longitudinal administrative data. Geographic school districts with greater than 1000 students are considered large. Math and reading achievement are standardized within test subject, grade, and year to the overall state distribution scores. Math and reading achievement are observed for grades 3 to 8; all other variables are observed for grades K-12. A full list of control districts is reported in table S2. All variables are from the 2013–2014 academic year.

	All districts	All large districts	Potential control districts	Flint
	Mean (SD)	Mean (SD)	Mean (SD)	Mean (SD)
Math achievement	−0.01	0.01	−0.34	−0.51
	(0.30)	(0.30)	(0.20)	(.)
Reading achievement	0.03	0.04	−0.31	−0.53
	(0.25)	(0.25)	(0.18)	(.)
Fraction special needs	0.15	0.14	0.15	0.15
	(0.04)	(0.03)	(0.02)	(.)
Fraction school days attended	0.95	0.95	0.94	0.91
	(0.03)	(0.01)	(0.01)	(.)
Fraction female	0.48	0.49	0.49	0.49
	(0.04)	(0.01)	(0.01)	(.)
Fraction Black	0.08	0.10	0.40	0.76
	(0.16)	(0.17)	(0.26)	(.)
Fraction Hispanic	0.06	0.06	0.12	0.04
	(0.08)	(0.08)	(0.14)	(.)
Fraction economically disadvantaged	0.51	0.49	0.75	0.89
	(0.18)	(0.19)	(0.11)	(.)
Fraction limited english proficiency	0.03	0.03	0.09	0.03
	(0.06)	(0.06)	(0.11)	(.)
Fraction attending charter schools	0.04	0.04	0.13	0.31
	(0.09)	(0.06)	(0.11)	(.)
Fraction attending administrative district	0.79	0.83	0.69	0.45
	(0.18)	(0.11)	(0.14)	(.)
Enrollment	2883	3947	7,496	16210
	(6083)	(7007)	(15,922)	(.)
Number of districts	548	362	54	1

#### Demeaned SCM

To isolate the causal effect of the FWC, we use an SCM approach that compares Flint to other Michigan districts over time. SCM was first developed by Abadie and Gardeazabal (*64*) and Abadie *et al.* (*65*). It is a longitudinal matching estimator designed to be used in situations with a single treated unit and a relatively small number of potential control units. SCM uses pretreatment outcome data to identify the weighted average of control districts that most closely approximates the treated unit. This approach can reduce bias through improved pretreatment fit and allow for a more transparent counterfactual selection process compared to other causal inference strategies using panel data.

We implement a recent extension of SCM known as demeaned, or intercept-shift, synthetic control. This approach involves first subtracting each treatment and control unit’s pretreatment outcome mean from all pre- and posttreatment observations (conceptually similar to a unit fixed effect) and then fitting a classic SCM model on those residuals (*66*). The demeaned SCM, similar to the classic difference-in-differences model, compares only changes over time (rather than average cross-sectional differences) between treated and control units.

While Flint’s mean academic outcomes are extreme outliers in the overall state distribution, its trends resemble many other districts that lie closer to the convex hull of control outcomes; thus, demeaning drastically increases our common support between Flint and potential control units. At the same time, demeaning does not stretch our relatively short panel of pretreatment outcomes too thin, as would more complicated extensions, such as incorporating time weights, outcome modeling, or machine learning.

The demeaned SCM that we implement is identical to several synthetic control extensions recently suggested in the literature. For example, our approach is equivalent to using augmented SCM in the specific case where each unit’s pretreatment mean is used as a covariate with the coefficient constrained to be equal to one (*13*). Our approach is also identical to fitting synthetic difference-in-differences with uniform time weights and a unit intercept (*14*). Hence, our demeaned SCM estimator can be identically expressed as a weighted difference-in-differences estimator. Suppose we have a panel of data with *N* unique districts *i* over time *t*. Flint, our treated district, has *i* = 1. Note that we follow the notation used in (*13*) and use *X_it_* that represents a district’s the outcome variable in the pretreatment period and *Y_it_* in the posttreatment period (see the Supplementary Materials for more details on our data structure and notation). Our estimating equation is

$$\mathrm{ATT}=\left({\bar{Y}}_{1}-{\bar{X}}_{1}\right)-[\sum_{i=2}^{N}w_{i}({\bar{Y}}_{i}-{\bar{X}}_{i})]$$ (1)

where *ATT* is the average treatment effect on the treated, ${\bar{Y}}_{i}$ is the average posttreatment educational outcome for district *i* in time *t*, ${\bar{X}}_{i}$ is the average pretreatment educational outcome for district *i*, and *w_i_* is the synthetic control weight for district *i*.

Next, we define ${\bar{X}}_{\text{syn}}$ and ${\bar{Y}}_{\text{syn}}$ as the average pretreatment and posttreatment outcomes of the synthetic Flint.

$$\begin{array}{c} {\bar{Y}}_{\text{syn}}=\sum_{i=2}^{N}w_{i}{\bar{Y}}_{i}\\ {\bar{X}}_{\text{syn}}=\sum_{i=2}^{N}w_{i}{\bar{X}}_{i} \end{array}$$ (2)

where ${\bar{Y}}_{\text{syn}}$ is the average posttreatment educational outcome of Flint’s synthetic control and ${\bar{X}}_{\text{syn}}$ is the average pretreatment educational outcome of Flint’s synthetic control.

It is then possible to rewrite Eq. 1 as

$$\mathrm{ATT}=\left({\bar{Y}}_{1}-{\bar{X}}_{1}\right)-\left({\bar{Y}}_{\text{syn}}-{\bar{X}}_{syn}\right)$$ (3)

While early versions of SCM balanced both lagged outcomes and covariates, we follow the recent literature (*13*, *14*) and choose to balance only lagged outcomes. One potentially unsatisfying aspect of existing SCM is that in the standard approach each implementation is specific to only a single outcome (e.g., synthetic Flint for math achievement may be composed of different control districts than synthetic Flint for reading achievement). We address this multiple outcome issue by finding a single set of SCM weights that simultaneously balances all four of our educational outcomes in the pretreatment period. Using more pretreatment data to identify our SCM weights also helps maximize their stability and thereby increase the precision of our analysis. The mathematical details of weight estimation are provided in the Supplementary Materials.

We obtain weights, treatment effect estimates, and SEs using the publicly available R package augsynth. The SEs are heteroskedasticity-consistent SEs for panel data settings using the R package sandwich (*67*) with variance estimated via the jackknife (*68*). A desirable feature of these empirical SEs is that they incorporate uncertainty created by the weight selection process in addition to uncertainty due to imperfect fit in the pretreatment period.

#### SCM robustness

We probe the robustness of our SCM results in a variety of ways. One concern is that the FWC might have induced selective student mobility and produced compositional changes in Flint, thereby biasing treatment effect estimates. To help address this concern, we construct a new district-year panel using invariant district assignment. Roughly two-thirds of Flint student attrition is within-state (see fig. S2), meaning that most students who leave Flint are nonetheless observed in other Michigan public schools. Under this invariant district assignment approach, a student is permanently assigned to a geographic school district using their home address in the 2013–2014 school year. Therefore, a student that moves from Flint to another Michigan district following the FWC will nonetheless contribute to the Flint average in these later years. We re-estimate our SCM using this new district-year panel. While such a strategy can be useful for reducing potential bias from selective student mobility, using only students who were observed in the 2013–2014 school year restricts our sample size and reduces statistical power (for instance, consider students who graduated before 2013–2014 or who entered kindergarten after 2013–2014). As an additional robustness check related to district mobility, we decompose our treatment effect estimates using a student’s (im)mobility status (see more details below).

As second concern is that our SCM results may be sensitive to the specific pool of control districts used. We test robustness on this front by fitting SCM models on two additional district samples: ∩75 and ∪95 (Fig. 3, B and C, respectively). Our ∩75 sample has a similar number of districts (*51*) as our primary sample (*54*) but instead requires the control districts to be high in both fraction Black and high in fraction economically disadvantaged. Our ∪95 is constructed similar to our primary sample but instead restricts to a narrower set of districts (*N* = 26) that are more similar to Flint using the 95th percentile (rather than the 90th percentile) of fraction Black and high in fraction economically disadvantaged.

A final concern is our results are sensitive to the fact that we fit a single SCM model on all four outcomes simultaneously. To address this, we fit six additional synthetic control models: each outcome on its own (single outcome SCM), as well as math achievement/reading achievement jointly and special needs/attendance jointly (double outcome SCM). Fitting an SCM model on just one or two outcomes (compared to all four) serves to increase pretreatment fit.

#### SCM treatment effect decomposition

To better understand effect heterogeneity and potential mechanisms, we decompose our estimated treatment effects (presented in Table 2) by five student-level characteristics: gender, grade, socioeconomic status, district mobility, and home service line material. To do so, we implement a novel and intuitive SCM treatment effect decomposition. Let the separate average treatment effects across some dichotomous subgroup *s* be expressed as

$$\begin{array}{c} {\mathrm{ATT}}^{s=0}=\left({\bar{Y}}_{1}^{s=0}-{\bar{X}}_{1}^{s=0}\right)-\left({\bar{Y}}_{syn}-{\bar{X}}_{syn}\right)\\ {\mathrm{ATT}}^{s=1}=\left({\bar{Y}}_{1}^{s=1}-{\bar{X}}_{1}^{s=1}\right)-\left({\bar{Y}}_{syn}-{\bar{X}}_{syn}\right) \end{array}$$ (4)

where *ATT*^*s* = 0^ is average treatment on the treated for Flint students with *s* = 0 and *ATT*^*s* = 1^ is the average treatment on the treated for Flint students with *s* = 1.

**Table 2.** Synthetic control weights and effect estimates. This table displays results from a synthetic control model using a sample of geographic school districts taken from the Michigan Department of Education’s longitudinal administrative data base. Math and reading achievement are standardized within test subject, grade, and year to the overall state distribution scores. Math and reading achievement are observed in only grades 3 to 8, whereas all other variables as observed in grades K-12. Average treatment effects estimates are displayed with jackknifed SEs in parentheses below. Individual outcome RMSE values are displayed in the specific units of each outcome (e.g., SD of the overall Michigan distribution of achievement, proportion for special needs, etc.), while the overall RMSE value is calculated after standardizing each variable within our analytic sample.

District weights		
District code	District name	Weight
14020	Dowagiac Union School District	0.15
25240	Beecher Community School District	0.04
35040	Whittemore-Prescott Area Schools	0.08
63250	Oak Park School District	0.19
72020	Houghton Lake Community Schools	0.09
80090	Bloomingdale Public School District	0.01
81070	Lincoln Consolidated School District	0.15
82060	Hamtramck School District	0.13
82120	River Rouge School District	0.10
82430	Van Buren Public Schools	0.05
Sum		1.00
**Estimates**		
	Pre-RMSE	Treatment effect
Math achievement	0.0216	−0.1439
		(0. 0649)
Reading achievement	0.0096	−0.0046
		(0.0215)
Special needs	0.0030	0.0124
		(0.0050)
Attendance	0.0034	−0.0018
		(0.0046)
Overall (scaled)	0.0677	

Notice that both *ATT*^*s* = 0^ and *ATT*^*s* = 1^, similar to the overall average treatment effects, are obtained using a weighted difference-in-differences estimator. *ATT*^*s* = 0^ compares Flint students with *s* = 0 to the synthetic control, whereas *ATT*^*s* = 1^ compares Flint students with *s* = 1 to the synthetic control. In the Supplementary Materials, we show algebraically that it is possible to decompose the overall average treatment effect displayed in Eq. 3 into a weighted average of *ATT*^*s* = 0^ and *ATT*^*s* = 1^ plus an adjustment for the pre/post change in the fraction of Flint students belonging to each subgroup

$$\mathrm{ATT}={\bar{p}}_{1}^{Y}\mathrm{AT}T^{s=0}+\left(1-{\bar{p}}_{1}^{Y}\right)\mathrm{AT}T^{s=1}+\Delta {\bar{p}}_{1}\left({\bar{X}}_{1}^{s=0}-{\bar{X}}_{1}^{s=1}\right)$$ (5)

where ${\bar{p}}_{1}^{Y}$ is posttreatment average fraction of Flint students with *s* = 1, $\Delta {\bar{p}}_{1}$ is the pretreatment to posttreatment change in average fraction of Flint students with *s* = 1, ${\bar{X}}_{1}^{s=0}$ is the pretreatment average educational outcome among Flint students with *s* = 0, and ${\bar{X}}_{1}^{s=1}$ is the pretreatment average educational outcome among Flint students with *s* = 1.

In our case, $\Delta {\bar{p}}_{1}$ tends to be small, so the overall treatment effects are approximately equal to the weighted average of the underlying subgroup effects, *ATT*^*s* = 0^ and *ATT*^*s* = 1^. Because our goal is to decompose treatment effect estimates given a set of SCM weights, SEs to test whether effects significantly differ across subgroups can be obtained using two-way fixed effects regression models. We choose to focus on a dichotomous decomposition because introducing subgroup categories also introduces more terms to adjust for pre/post proportion changes, putting more distance between the overall SCM average treatment effect and the weighted average of the subgroup treatment effects. In addition, using a dichotomous decomposition avoids slicing our data too thinly.

We elected to use this particular decomposition, rather than either (i) fitting new SCM models on subsamples of Flint students or (ii) taking the difference between a given Flint subgroup and that same subgroup in the synthetic control, for a few different reasons. First, our preferred decomposition can be implemented even when some of the variables (such as service line material) are observed for Flint but not for the control districts. Second, differences between a given subgroup in Flint and that same subgroup in the synthetic control do not tend to cleanly average out to the overall treatment effects, as Flint and the synthetic control have mean differences in subgroup proportions for certain variables (such as socioeconomic status). Last, estimating an entirely new synthetic Flint for different subgroups is conceptually unsatisfying (in exactly the same way estimating a separate synthetic Flint for different outcomes variables is).

## RESULTS

**Fig. 4.**
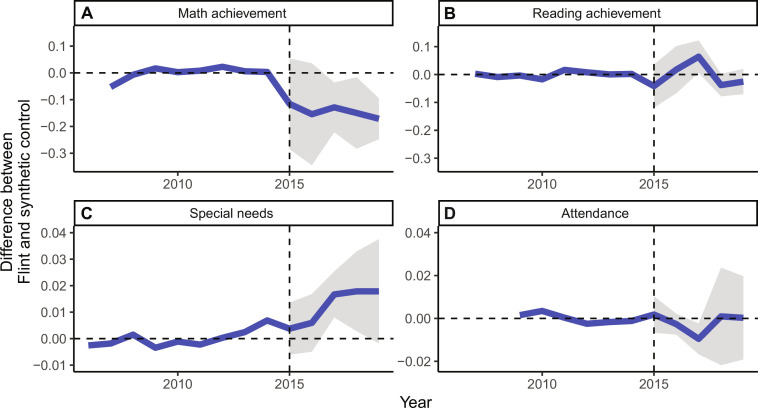
Synthetic control estimates of the effect of the FWC on student outcomes. Average treatment effect estimates of the causal effects of FWC on standardized math and reading academic achievement (**A** and **B**), fraction special needs (**C**), and fraction of school days attended (**D**) are plotted over time. 2015 is the first year post-treatment. A treatment effect was estimated for each year in the post-period (2015–2019). The grayed shaded area represents the 95% confidence interval of the treatment effect estimates. Synthetic Flint was constructed by taking a weighted average of the districts listed in Table 2.

Figure 4 presents the SCM estimates described above. The thick blue line measures the difference between student outcomes in Flint versus the synthetic control group. Starting in 2015, the blue line reflects the treatment effect of the FWC and the shaded gray area corresponds to the confidence interval around the estimate.

Table 2 lists the 10 control districts assigned a nonzero weight in the construction of the Flint counterfactual. Four districts play a large role in the comparison: Oak Park, Lincoln, Dowagiac, and River Rouge. River Rouge and Oak Park are small urban districts near Detroit with very high percentages of both low-income and Black students. Dowagiac is a predominantly white district (roughly 65%) located in the Southwest part of the state with very high poverty levels (about 75%). Lincoln is an urban district located near Ypsilanti, Michigan, roughly 45 min southwest of Detroit. While the selected comparisons are similar to Flint in these demographic characteristics, the most important test of the SCM model is how closely the outcome trends of the controls match Flint’s before the FWC. As one would hope, the blue line before 2014 is nearly zero, suggesting a very good match. The root mean square error of each outcome variable in the pretreatment period (pre-RMSE) provides a formal measure of model fit. As can be seen in Table 2, the pre-RMSEs are generally low, showing that even while balancing four outcomes simultaneously, we are able to provide good fit in the pretreatment period.

Our results suggest the FWC negatively affected student performance along several dimensions. Math achievement in Flint closely tracks the comparison districts from 2006 to 2014 but drops notably starting in 2015. The SCM estimates indicate a 0.14 SD decrease in student performance in math. The 0.14 SD effect on math achievement falls within the range of “medium” effect size according to recent standards for educational interventions (*69*). However, when one considers both the large size of the treated group and the fact that the effect is negative, math results might be considered “large”. We did not observe a significant detectable effect on reading achievement in our main analysis. This pattern of results is consistent with many other studies that suggest student math performance is more malleable to interventions than reading performances in the short-term (*70*). We find no impact on student attendance (or chronic absenteeism, presented in fig. S3).

Finally, Fig. 4 the FWC led to a 1.2 percentage point (8%) increase in the proportion of students with a documented special educational need. It is possible that the heightened attention on Flint schoolchildren during the crisis led to greater screening, which in turn increased special needs diagnosis rates while leaving underlying population characteristics unchanged. For instance, lawsuits brought by the American Civil Liberties Union and the Education Law Center against the City of Flint spurred efforts to identify children in need of academic support. On the other hand, the concurrent reduction in math performance, which is not subject to bias due to increased screening, suggests that underlying needs in the population likely increased as well. We suspect the observed increase reflects some combination of both forces, although it is difficult to know the exact magnitude of each.

**Fig. 5.**
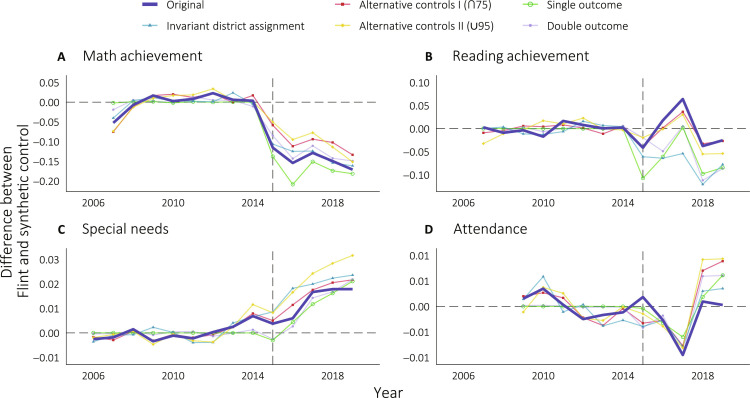
Visualizing synthetic control robustness checks. Average treatment effect estimates of the causal effects of FWC on standardized math and reading academic achievement (**A** and **B**), fraction special needs (**C**), and fraction of school days attended (**D**) are plotted over time. 2015 is the first year post-treatment. Results from our primary synthetic control model is displayed in dark blue, and the other colored lines represent results from alternative synthetic control models used as robustness checks. See Materials and Methods for a detailed description of each alternative synthetic control model.

Next, we consider the results of our various robustness checks. Figure 5 graphically displays our primary SCM results overlaid on the five alternative models for each outcome, and Table 3 reports the estimated average treatment effects and corresponding SEs. Across all the specifications, our key findings—a decrease in math achievement and an increase in special needs—remain largely unchanged.

**Table 3.** Synthetic control robustness checks effects estimates. This table displays results from a synthetic control model using a sample of geographic school districts taken from the Michigan Department of Education’s longitudinal administrative data base. Math and reading achievement are standardized within test subject, grade, and year to the overall state distribution scores. Math and reading achievement are observed in only grades 3 to 8, whereas all other variables as observed in grades K-12. Average treatment effects estimates are displayed with jackknifed SEs in parentheses below.

	Original	Invariant district assignment	Alternative controls I (∩75)	Alternative controls II (∪95)	Single outcome	Double outcome
Math achievement	−0.1439	−0.1341	−0.1001	−0.0977	−0.1707	−0.1261
	(0.0649)	(0.0533)	(0.0073)	(0.0160)	(0.0606)	(0.0333)
Reading achievement	−0.0046	−0.0753	−0.0121	−0.0198	−0.0696	−0.0526
	(0.0215)	(0.0321)	(0.0287)	(0.0221)	(0.0816)	(0.0714)
Special needs	0.0124	0.0185	0.0152	0.0218	0.0101	0.0106
	(0.0050)	(0.0055)	(0.0022)	(0.0055)	(0.0065)	(0.0057)
Attendance	−0.0018	−0.0016	0.0004	0.0010	−0.0003	−0.0006
	(0.0046)	(0.0013)	(0.0031)	(0.0034)	(0.0015)	(0.0039)

The magnitude of the decrease in math achievement ranges from −0.10 SD (∪95 sample) to −0.17 SD (single outcome SCM), and the magnitudes of the increase in special needs ranges from 1.0 percentage points (single outcome SCM) to 2.2 percentage points (U95 sample). SCM weights for our invariant district assignment, ∩75 sample, and U95 sample models can be found in table S4.

Next, we apply our subgroup decomposition to math achievement and special needs status; results are displayed in Table 4. Our decomposition uses regression-based SEs that do not account for the uncertainty that results from the weight selection procedure and therefore cannot be reliably used to test whether a subgroup effect is statistically significant when the overall effect was itself insignificant in the SCM model. For this reason, we omit displaying decomposition results for reading achievement and attendance.

**Table 4.** Synthetic control effect decompositions. This table displays results from a two-group decomposition of observed treatment effects on math achievement and special needs status across five student-level characteristics. See the Synthetic Control Methods section of the Supplementary Materials for a detailed description the decomposition approach. SEs are estimated using two-way fixed effects regression models. The average proportion of Flint students in each given subgroup category is denoted by *p*. Results (i.e., *P* values) from a from a test of the equivalence in effect size across the two groups are displayed at the bottom of each panel. Note that service line material information was only available for a subsample of address-matched Flint students. Observed Δ${\bar{p}}_{1}$ for the various subgroup variables are as follows: gender = 0.002, grade = 0.034, socioeconomic status = 0.085, service line material = 0.064, and district mobility = 0.057.

	Math achievement	Special needs
**Gender**		
Male [*p* = 0.51]	−0.151	0.0210
	(0.0150)	(0.00377)
Female [*p* = 0.49]	−0.137	0.00418
	(0.0125)	(0.00190)
* P*(male = female)	0.462	≤0.001
**Grade**		
Grade 5 [*p* = 0.48]	−0.193	0.0113
	(0.0132)	(0.00283)
Grade 6 [*p* = 0.52]	−0.0895	0.0137
	(0.0178)	(0.00380)
* P*(grade ≤ 5 = grade ≥ 6)	≤0.001	0.610
**Socioeconomic status**		
Below medium [*p* = 0.59]	−0.146	0.00736
	(0.0161)	(0.00507)
Above median [*p* = 0.41]	−0.0850	0.0168
	(0.0133)	(0.00535)
* P*(below median = above median)	0.003	0.200
**Service line material**		
Copper [*p* = 0.6]	−0.161	0.0195
	(0.0184)	(0.00433)
Lead [*p* = 0.4]	−0.191	0.0212
	(0.0259)	(0.00527)
* P*(copper = lead)	0.344	0.796
**District mobility**		
Immobile [*p* = 0.6]	−0.140	0.0171
	(0.0158)	(0.00333)
Mobile [*p* = 0.4]	−0.147	0.0103
	(0.0119)	(0.00313)
* P*(immobile = mobile)	0.747	0.139

In Table 4, we see that effect estimates on math achievement are highly similar across gender. However, our effect estimates on special needs status significantly differ for boys and girls (*P* < 0.001); whereas boys experienced a 2.1 percentage point increase, girls experienced only a 0.4 percentage point increase in special needs classification. This amounts to meaningful heterogeneity even when considering the baseline gender differences in special needs classification before the FWC (19.3% of male students and 10.1% of female students): Boys experienced a 10.9% increase in special needs, whereas girls experienced just a 4.0% increase.

Turning to grade, we find evidence that effects on math achievement significantly differ (*P* < 0.001). Students in grades 3 to 5 experienced a 0.19 SD decrease in math achievement, whereas students in grades 6 to 8 experienced a 0.09 SD decrease. We find no evidence that effects on special needs classification varied by grade level. In addition, we find evidence of socioeconomic effect heterogeneity for math achievement (*P* = 0.003) but not for special needs status. While Flint students with socioeconomic status below the median experienced a 0.15 SD decline in math achievement, students above the median experienced only a 0.09 SD decline.

**Fig. 6.**
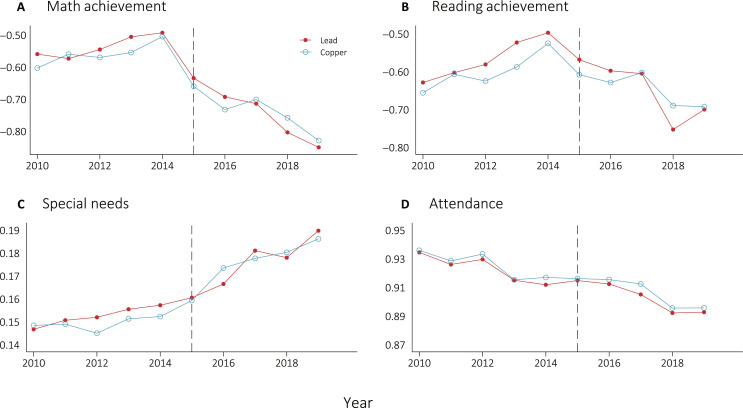
Mean educational outcomes over time in Flint by service line material. This figure displays descriptive trends in the mean academic outcomes for the Flint geographic district from 2010–2019. Education data is taken from the Michigan Department of Education’s longitudinal administrative data base. The blue lines display students living in homes with copper service lines, while the red lines display students living in homes with lead service lines. Service line material data was collected by the City of Flint’s service line inspection and replacement program implemented following the crisis. The black dotted vertical line represents time that the FWC begins (i.e., the first year in the post-period). Math and reading achievement (**A** and **B**) are observed in only grades 3 to 8, whereas special needs (**C**) and attendance (**D**) are observed in grades K-12.

We see that effect estimates do not meaningfully differ across service line material. Figure 6 displays the highly similar trends in achievement outcomes across those with lead and copper service lines. A detailed difference-in-differences analysis using student-level data to assess the impact of lead service lines, available in the Supplementary Materials, yields substantively similar results [see also (*71*)].

Lastly, we turn to district mobility. There are no differences in the estimated effects for immobile and mobile students, though results for mobile students are difficult to substantively interpret due to the mechanically changing sample over time. Nonetheless, considering the effects on immobile students, which necessarily cannot be driven by selective mobility or attrition, serves as a useful robustness check. Reassuringly, estimated effects for the immobile subgroup are nearly identical to our overall SCM effects for math achievement and, if anything, slightly larger in magnitude for special needs status (1.7 percentage points compared to 1.2 percentage points).

## DISCUSSION

We find substantial negative effects of the FWC on the academic outcomes of children living in Flint. Using SCM models which compare Flint to observationally equivalent school districts in Michigan, we find compelling evidence that the FWC reduced student math achievement and increased the rate of special needs classification. However, using a treatment effect decomposition, we find little difference in the academic trajectories of Flint children living in homes with lead service lines. What might explain these perhaps unexpected results?

One potential explanation could be that a child’s home service line material is a noisy proxy for their true risk of lead exposure during the FWC. Children consume a portion of their water outside the home [e.g., at school, as examined in (*72*)], and service lines are not the only source of lead in water systems—lead fixtures and lead solder also play a role. In addition, if children consume water at their neighbors’ houses or if one child’s educational outcomes affect the outcomes of their peers, treatment spillovers may be introduced. Thus, there may have been impacts of lead exposure during the FWC on academic outcomes among the school-age children in our study that went undetected.

However, by leveraging prior research and focusing specifically on math achievement, we can see that the expected decrease as a result of lead exposure cannot account for the total math effects estimated in our SCM analysis. In the Supplementary Materials, we use past benchmarks to show that we would expect a 0.017 SD decrease in math scores as a result of direct lead effects observed during the FWC, an order of magnitude smaller than the 0.14 SD decrease in math achievement observed. Moreover, only 40% of Flint children were exposed to lead service lines at their residence, meaning direct lead effects would attenuate in the SCM analysis. Thus, both theoretically and empirically, lead pathways are insufficient to fully explain the between-district results.

While the precise mechanisms of the effects observed in this study remain largely unknown, they are unlikely to be driven by lead poisoning alone. At first blush, the FWC may appear as predominantly an environmental health catastrophe, but results show that there is more to the story. The complex community-level process that created a crisis surrounding the actual lead exposure played a substantial role, in addition to whatever direct health effects lead did have. That is, we would expect the effects of only lead to be smaller than the overall effects of the FWC. The FWC affected all children living in Flint, not only those children who experienced elevated lead exposure (just as the COVID-19 pandemic affected children who never contracted the virus). These broad effects may be linked to the psychosocial impacts of a child experiencing a crisis or may have operated through other non-lead pathways (for instance, higher teacher turnover or increases in local crime). While it is unclear why we see achievement effects in math but not reading, math scores have been shown to be more sensitive to other short-term mechanisms in middle childhood, such as summer learning loss (*70*). Similarly, COVID-19 pandemic-related learning losses were shown to be three times larger for math achievement than for reading achievement (*51*).

In addition, that we do not observe average treatment effects on attendance is surprising, given the social upheaval associated with the crisis. However, there was a significant negative effect on attendance (and increase in chronic absenteeism) in 2016, the year that the story of the FWC broke nationally. One possible explanation is that the effects on attendance were brief and perhaps largely abated by the increased resources and attention Flint received in the following years. Another possibility is that the accuracy of our attendance data, which (unlike achievement and special needs) is collected daily by teachers, was itself affected by the FWC.

Our decomposition analyses identify meaningful effect heterogeneity across gender, grade, and socioeconomic status. We find that the negative effect on math achievement was larger for students below (rather than above) the median of Flint’s socioeconomic distribution and for students in grades 3 to 5 (rather than grades 6 to 8). That socioeconomic differences, even in an incredibly high poverty context such as Flint (where about 9 of 10 students qualify as economically disadvantaged), that stratifies the impact of disruptive events is a new and important contribution to the literature. It also highlights the coarseness of existing poverty measures used by schools systems and the need for improved data and tools for quantifying socioeconomic disadvantage. That younger students that bore the brunt the FWC’s negative effects on math achievement suggests that there is likely ongoing opportunities for remediation.

The effects on special needs classification, on the other hand, were driven largely driven by effects on male students, meaning that the FWC served to exacerbate already substantial differences in special needs classification across gender. Given that we observed highly similar effects on math achievement for boys and girls, it is possible be that the FWC did not, in fact, induce gender-specific changes in objective special needs qualification; instead, these different effects on special needs classification may represent biases in existing identification and evaluation processes.

A lingering question is why home service line material did not impact how the crisis affected school-age children. One possibility is that many Flint residents stopped drinking tap water at home immediately after the switch. Alternatively, it may be that those in houses with lead pipes did consume elevated levels of lead, but this exposure was not high enough to cause measurable academic impairments in school-age children. Previous quasi-experimental studies focus on children exposed to lead around 1 to 3 years of age, whereas the average age of exposure of children for whom we observe math and reading achievement is about 6 years old: beyond their period of greatest sensitivity to the neurotoxic effects of lead.

Should the broad effects observed in Flint generalize to other lead-in-water crises? There are reasons to be cautious; unlike other water crises, the change in Flint happened discretely and was accompanied by a change in taste and discoloration, making it highly noticeable to the city’s populace and likely that Flint citizens substituted away from contaminated tap water at greater rates than other crises (which would decrease the lead effects). At the same time, the magnitude of cover-up and ensuing scandal was large and received prolonged national attention, meaning that Flint citizens were likely more aware of the crisis than people exposed to lead in tap water elsewhere (thereby heightening the social and psychological mechanisms).

Nonetheless, documenting the Flint case is important in and of itself, and it also serves as a meaningful demonstration that the ways in which such events may affect citizens are not necessarily straightforward; one cannot treat these crises as strictly medical phenomena. Finally, our results suggest that we currently substantially underestimate the costs of the FWC. Existing estimates of the health effects of the crisis, ranging from 50 to 400 million dollars (*2*, *73*), use only the lead effects. This study ought to draw attention to the large potential costs of these water crises and motivate preventative measures, which by comparison are cheap. Tragically, the Flint water switch was intended to save just 5 to 7 million dollars.

Our study has important limitations. First and foremost, we use school records that can only identify children living in Flint when they enter school. Therefore, we cannot speak to the educational consequences of the FWC on infants or young children, who were likely more substantially affected than the school-age children we observe. Another limitation is that our analysis is restricted to specific educational outcomes captured in state administrative data, and we are therefore unable to detect impacts on a variety of important behaviors and skills. Moreover, while using service line inspection data helps us to separate the direct health effects of lead from the broader effects of the resulting crisis, we cannot say precisely what pathways any indirect effects may operate through. Lastly, the substantial public health response to the crisis likely mitigated potential negative effects, suggesting that our between-district results may underestimate the impact of the crisis alone. Local organizations in Flint, supported by state, federal, and foundation funding, responded to the crisis by markedly expanding the set of social, medical, and educational services available to children in Flint, ranging from positive messaging campaigns to free childcare to literacy programs.

Flint is just one piece of a broader puzzle regarding the ways in which lead plumbing undergirds processes that produce health and social burdens in the United States today. As the risk of exposure from lead paint continues to fall with the success of many public health interventions, policymakers may do well to begin to shift their attention back toward understanding lead plumbing, and the community crises that its presence can help create.

## Supplementary Materials

### This PDF file includes:

Supplementary Text Figs. S1 to S4 Tables S1 to S10 References
