# Supplementary material for: The Effects of the Flint water crisis on the educational outcomes of school-age children
Source: Sci Adv. 2024 Mar 13;10(11):eadk4737. doi: 10.1126/sciadv.adk4737 (PMC10936875; doi:10.1126/sciadv.adk4737)
Supplement: Supplementary file 1 — Supplementary Text Figs. S1 to S4 Tables S1 to S10 References [file sciadv.adk4737_sm.pdf]

Science Advances
AAAS

Supplementary Materials for

The effects of the Flint water crisis on the educational outcomes of school-age children

Sam Trejo *et al.*

Corresponding author: Sam Trejo, samtrejo@princeton.edu

Sci. Adv. **10**, eadk4737 (2024)
DOI: 10.1126/sciadv.adk4737

This PDF file includes:

Supplementary Text
Figs. S1 to S4
Tables S1 to S10
References

SUPPLEMENTARY INFORMATION

The Effects of the Flint Water Crisis on the Educational Outcomes of School-Age Children

Trejo, Yeomans-Maldonado, & Jacob 2024

THE EXPECTED EDUCATIONAL IMPACTS OF LEAD ON FLINT CHILDREN

How would we expect the amount of increased lead exposure that resulted from the Flint Water Crisis to impact educational outcomes? There are a few studies that provide recent and rigorous evidence regarding the effects of blood lead on academic achievement and intelligence. Aizer et al. 2018 [21] uses an instrumental variables strategy, whereas Lanphear et al. 2005 [74] and Reuben at al. 2017 [10] are simple linear regression (controlling for confounding factors such as parental cognition and childhood socioeconomic status). The relevant results from these three studies are displayed in Table S1. We omit Billings & Schnepel 2018 [20] because they leverage lead reductions that results from a broad public health program that may have had its additional, non-lead driven effects on educational development.

In Aizer et al. 2018 [21], the authors use two different instrumental variables strategies to quantify the causal effects of blood lead, measured in early childhood (i.e., below the age of 6), on third grade math and reading test scores using administrative data from Rhode Island. The first strategy uses one childhood measure of blood lead as an instrument for another from the same child, thereby reducing measurement error (Blood Lead IV). The second strategy uses the timing and geographic roll-out of a lead-free home certificate program as an instrument for a child's blood levels (Certificate IV). The second stage of both approaches estimates the effect of (predicted) blood lead on both math and reading achievement. All point estimates are negative and of the same order of magnitude but estimates from the Blood Lead IV are much more precise than estimates from the Certificate IV.

A second relevant study is Reuben et al. 2017 [10]. Here, the authors use rich longitudinal data from New Zealand to estimate the association between blood lead measured at age 11 on IQ in adulthood (age 38). They provide unconditional estimates as well as estimates from regression models that control for sex, maternal IQ, IQ at age 11, and children's socioeconomic status. The association between blood lead and IQ attenuates only slightly when controls are included.

A third study, Lanphear et al. 2005 [74], presents a pooled international analysis that measures the association between blood lead and children's IQ. They find find nonlinear effects of lead; that is, the marginal blood lead effects are larger for children with less lead in their body than children with more lead in their body. For this reason, we focus on the study's estimates from the lower end of the lead distribution (<10 ug/dL), where most Flint children fell both before and after crisis. Using their preferred estimate, Lanphear et al. 2005 [74] observes a "decline of 6.2 IQ points (95% CI, 3.8-8.6) for an increase in blood lead levels from < 1 to 10 pg/d." Dividing 6.2 by 10 yields the effect of a one ug/dL change in blood lead on unstandardized IQ points per entails an average (0.62). Finally, if we divide 0.62 by the standard deviation of the IQ measure in their study (19.2), we are left with the effect of a one ug/dL change in lead on standardized IQ (.032).

In summary, the results from these three studies, converge surprisingly well; a one microgram per deciliter (ug/dL) increase in blood lead is associated with approximately 0.03 SD reduction in cognitive ability, math achievement, and reading achievement. Given that children experienced roughly a 0.5 ug/dL average increase in blood lead following the Flint Water Crisis, we would expect only a .017 SD decrease in math scores *as a result of lead effects*. In the education literature, a .017 SD effect is quite small [69]. Lead is also known to have effects on behavioral outcomes, which we attempt to index through special needs status and daily attendance, though the expected effects on these outcomes are unknown.

SYNTHETIC CONTROL METHODS

Notation and Data Structure

Suppose we have a panel of data with districts i over time t. Flint, our treated district, has $i = 1$. We observe a total of T time periods of some educational outcome Y_{it} for N unique school districts. The final pretreatment year is t^* such that $1 < t^* < T$. Here, $T = 14$ (i.e. we have yearly observations ranging from $2006 - 2019$), $t^* = 9$ (i.e. 2014), and $N = 55$ (i.e. Flint and 54 control districts).

We begin with our data structured as the following $N \times T$ matrix:

$$\begin{matrix} Flint \\ Detroit \\ \vdots \\ Lansing \end{matrix} \begin{bmatrix} Y_{1,1} & Y_{1,2} & \cdots & Y_{1,t^*} & Y_{1,t^*+1} & \cdots & Y_{1,T} \\ Y_{2,1} & Y_{2,2} & \cdots & Y_{2,t^*} & Y_{2,t^*+1} & \cdots & Y_{2,T} \\ \vdots & & & & & & \vdots \\ Y_{N,1} & Y_{\mathrm{N},2} & \cdots & Y_{N,t^*} & Y_{N,t^*+1} & \cdots & Y_{N,T} \end{bmatrix}$$

(S1)

Y_{it}: Educational outcome for district i in time t
T: Scalar for total number of time periods observed
N: Scalar for total number of unique districts in our data
t^*: Final year t before treatment begins

To aid in the exposition of our methodology, for the remainder of the paper we partition pretreatment outcome observations (i.e. $t \leq t^*$) into X_{it} and use Y_{it} to refer solely to posttreatment observations (i.e. $t > t^*$).

$$\begin{bmatrix} X_{1,1} & X_{1,2} & \cdots & X_{1,t^*} & Y_{1,t^*+1} & \cdots & Y_{1T} \\ X_{2,1} & X_{2,2} & \cdots & X_{2,t^*} & Y_{2,t^*+1} & \cdots & Y_{2T} \\ \vdots & & & & & & \vdots \\ X_{\mathrm{N},1} & X_{N,2} & \cdots & X_{N,t^*} & Y_{N,t^*+1} & \cdots & Y_{NT} \end{bmatrix}$$

(S2)

Conceptualizing Treatment Effects

Using the potential outcomes framework [75], in order to calculate the causal effect of the Flint Water Crisis on some educational outcome Y_{it}, we must identify the difference between the potential outcome that was observed in Flint, $Y_{1t}(1)$, and the potential outcome that we would have observed if Flint had not experienced its water crisis, $Y_{1t}(0)$.

$$ATT_t = Y_{1t}(1) - Y_{1t}(0)$$

$$ATT = \frac{1}{T - t^*} \sum_{t=1}^{T-t^*} ATT_t$$

(S3)

ATT_t: Average treatment on the treated of the Flint Water Crisis in year t
ATT: Overall average treatment on the treated of the Flint Water Crisis
$Y_{1t}(1)$: Potential outcome for Flint (i.e i=1) in year t when treated
$Y_{1t}(0)$: Potential outcome for Flint (i.e i=1) in year t when untreated

Of course, we do not observe both $Y_{it}(1)$ and $Y_{it}(0)$ for any district in our panel. Instead, we observe $Y_{it}(1)$ for Flint and $Y_{it}(0)$ for all our control districts. Thus, we are left needing a reliable way to combine $Y_{it}(0)$ for the 54 control districts to approximate Flint's potential outcome in the absence of treatment, $Y_{1t}(0)$.

In many panel data settings, difference-in-differences methods are a credible way to obtain $\hat{Y}_{1t}(0)$, an estimate of $Y_{1t}(0)$, and thereby estimate the causal effects of an event like the Flint Water Crisis. Nonetheless, there exist some challenges to estimating difference-in-differences with a single treated unit remain[76], [77]. However, finding a valid counterfactual for Flint is quite difficult. Even setting aside the water crisis, as a waning mid-size city with residents who are disproportionately black and poor, Flint is unique. No districts in our panel readily support the common trends assumption required by differences-in-differences models.

Estimating Weights

We construct the vector of synthetic control weights, $\boldsymbol{w}$**,** by solving for the values that minimizes the squared distance between demeaned pretreatment outcomes for Flint and demeaned pretreatment outcomes for the other districts.

$$\boldsymbol{w} = \begin{bmatrix} w_2 \\ w_3 \\ \vdots \\ w_N \end{bmatrix}$$

(S4)

$\boldsymbol{w}$: $N - 1 \times 1$ vector of basic synthetic control weights w_i from $\text{i} = 2, \dots, \text{N}$

Specifically, we solve:

$$\min_{\boldsymbol{w}} \sum_{t=1}^{t*} [(X_{1t} - \bar{X}_1) - \sum_{i=2}^{N} (X_{it} - \bar{X}_i)\, w_i]$$

$$\text{subject to} \quad \sum_{i=2}^{N} w_i = 1$$

$$w_i \geq 0, i = 2, \dots, N$$

(S5)

However, our problem is, in practice, slightly more complicated. We identify a single set of synthetic control weights that simultaneously balances all four of our educational outcomes in pretreatment period. Let us denote each outcome with j, where J is the total outcomes. For every outcome j, we have a unique $N \times T$ matrix described in Equation S2. Thus, we can solve for the values of $\boldsymbol{w}$ that simultaneously minimize the squared distance between all J demeaned pretreatment outcomes for Flint and all J demeaned pretreatment outcomes for control districts. We first normalize each outcome by dividing it by its standard deviation so that differences in scaling do not influence the optimization.

$$\min_{\boldsymbol{w}} \sum_{j=1}^{J} \sum_{t=1}^{t*-1} [(X_{1tj} - \bar{X}_{1j}) - \sum_{i=2}^{N} \left(X_{itj} - \bar{X}_{ij}\right) w_i] \tag{S6}$$

Synthetic Control Decomposition

To illustrate our treatment effect decomposition, begin with Equation 3 from the main manuscript:

$$ATT = [\bar{Y}_1 - \bar{X}_1] - [\bar{Y}_{syn} - \bar{X}_{syn}]$$

Any posttreatment average educational outcome for a given district is simply a weighted average of the two subgroups, so we can simply substitute in $\bar{Y}_1 = (\bar{p}_1^Y)\bar{Y}_1^{s=0} + (1 - \bar{p}_1^Y)\bar{Y}_1^{s=1}$. This leaves us with:

$$ATT = [(\bar{p}_1^Y)\bar{Y}_1^{s=0} + (1 - \bar{p}_1^Y)\bar{Y}_1^{s=1} - \bar{X}_1] - [\bar{Y}_{syn} - \bar{X}_{syn}]$$

The same applies to pretreatment average educational outcomes, so we can substitute in $X_1 = (\bar{p}_1^X)\bar{X}_1^{s=0} + (1 - \bar{p}_1^X)X_1^{s=1}$:

$$ATT = [(\bar{p}_1^Y)\bar{Y}_1^{s=0} + (1 - \bar{p}_1^Y)\bar{Y}_1^{s=1} - (\bar{p}_1^X)\bar{X}_1^{s=0} - (1 - \bar{p}_1^X)X_1^{s=1}] - [\bar{Y}_{syn} - \bar{X}_{syn}]$$

Next, we algebraically rearrange so the the subgroup posttreatment and posttreatment terms are next to one another:

$$ATT = [(\bar{p}_1^Y)\bar{Y}_1^{s=0} - (\bar{p}_1^X)\bar{X}_1^{s=0} + (1 - \bar{p}_1^Y)\bar{Y}_1^{s=1} - (1 - \bar{p}_1^X)X_1^{s=1}] - [\bar{Y}_{syn} - \bar{X}_{syn}] \tag{S7}$$

Then, we define a new term, $\Delta\bar{p}_1$. $\Delta\bar{p}_1$ represents the change in the change in Flint's subgroup proportion from pretreatment period to the posttreatment period.

$$\Delta\bar{p}_1 \ = \bar{p}_1^Y - \bar{p}_1^X$$

By rearranging, we are left with:

$$\bar{p}_1^X \ = \bar{p}_1^Y - \Delta\bar{p}_1$$

We then can substitute $\bar{p}_1^X \ = \bar{p}_1^Y - \Delta\bar{p}_1$ back into Equation S7 above:

$$ATT = [(\bar{p}_1^Y)\bar{Y}_1^{s=0} - (\bar{p}_1^Y - \Delta\bar{p}_1)\bar{X}_1^{s=0} + (1 - \bar{p}_1^Y)\bar{Y}_1^{s=1} - (1 - \bar{p}_1^Y + \Delta\bar{p}_1)X_1^{s=1}] \ - [\bar{Y}_{syn} - \bar{X}_{syn}]$$

We algebraically rearrange:

$$ATT = [\bar{p}_1^Y(\bar{Y}_1^{s=0} - \bar{X}_1^{s=0}) + \Delta\bar{p}_1\bar{X}_1^{s=0} + (1 - \bar{p}_1^Y)(\bar{Y}_1^{s=1}) - \Delta\bar{p}_1 X_1^{s=1}] - \left[\bar{Y}_{syn} - \bar{X}_{syn}\right]$$

Again, we algebraically rearrange:

$$ATT = \bar{p}_1^Y(\bar{Y}_1^{s=0} - \bar{X}_1^{s=0}) + (1 - \bar{p}_1^Y)(\bar{Y}_1^{s=1}) - \Delta\bar{p}_1(\bar{X}_1^{s=0} - X_1^{s=1}) - \left[\bar{Y}_{syn} - \bar{X}_{syn}\right]$$

Finally, we multiply the synthetic control term on the far right by $1 = \bar{p}_1^Y + (1 - \bar{p}_1^Y)$:

$$ATT = \bar{p}_1^Y(\bar{Y}_1^{s=0} - \bar{X}_1^{s=0}) + (1 - \bar{p}_1^Y)(\bar{Y}_1^{s=1} - \bar{X}_1^{s=1}) - \Delta\bar{p}_1(\bar{X}_1^{s=0} - X_1^{s=1}) - [\bar{p}_1^Y + (1 - \bar{p}_1^Y)][\bar{Y}_{syn} - \bar{X}_{syn}]$$

We algebraically rearrange one last time:

$$ATT = \bar{p}_1^Y\big([\bar{Y}_1^{s=0} - \bar{X}_1^{s=0}] - [\bar{Y}_{syn} - \bar{X}_{syn}]\big) + (1 - \bar{p}_1^Y)\big([\bar{Y}_1^{s=1} - -\bar{X}_1^{s=1}] - [\bar{Y}_{syn} - \bar{X}_{syn}]\big) - \Delta\bar{p}_1(\bar{X}_1^{s=0} - X_1^{s=1})$$

Recall, from the main text, that $[\bar{Y}_1^{s=0} - \bar{X}_1^{s=0}] - [\bar{Y}_{syn} - \bar{X}_{syn}] = ATT^{s=0}$ and $[\bar{Y}_1^{s=1} - \bar{X}_1^{s=1}] - [\bar{Y}_{syn} - \bar{X}_{syn}] = ATT^{s=1}$. Each of these terms are simply difference-and-differences equations between the Flint subgroups and the overall synthetic control. This leaves us with:

$$ATT = \bar{p}_1^Y ATT^{s=0} + (1 - \bar{p}_1^Y) ATT^{s=1} + \Delta\, \bar{p}_1 (\bar{X}_1^{s=0} - \bar{X}_1^{s=1})$$

Thus, our overall average treatment effect is a weighted average of the within-subgroup treatment effects and a final parameter, $\Delta\, \bar{p}_1 (\bar{X}_1^{s=0} - \bar{X}_1^{s=1})$, which accounts for the change in subgroup proportions from the pretreatment to the posttreatment period.

WITHIN-FLINT DIFFERENCE-IN-DIFFERENCES ANALYSIS

In this section, we conduct a within-Flint analysis to better isolate the effects of lead exposure itself on student outcomes. In particular, we compare the educational trajectories of Flint children living in housing units that were exposed to different levels of lead contamination during the water crisis. We ask: How did Flint children who lived in homes with lead pipes fare compared to Flint children who lived in homes with copper pipes before and after the crisis? We begin by describing our student-level panel data and then discuss the DiD approach that we utilize.

Service Line Data

To identify households with the greatest risk of exposure to lead contamination, we use data on the materials of the water service lines running to individual buildings (we call this the "pipes data"). This data comes from service line inspections conducted by Flint's FAST Start program, a team of city and state officials tasked with managing lead service line replacement following the FWC. Academic researchers partnered with the FAST Start team to help with data management and refine the prediction of service line material [14], developing a web and mobile application where on-site contractors and public officials filled in information about service line work at each site. These researchers have since formed the company BlueConduit to continue their municipal lead service line prediction work. Our data was extracted for their database on January 8, 2020. In total, this data includes 24,646 unique parcels with valid inspections, representing most but not all occupied properties in Flint with active water accounts that met certain minimum criteria regarding house age.

The inspection data indicates the material used in both the public service line (i.e., the pipes running from the city water supply to the private home) and the private service line (i.e., the pipes running from the public lines into the specific unit and to the faucets within the home). In addition to lead, galvanized steel is considered a dangerous material for service lines that have at one point contained lead in the system because they have been known to capture small pieces of lead in the corrosion in their inner walls. For our main analysis, we follow the approach used by the FAST Start team and consider any home with lead, galvanized steel, or an unknown material in either the public or private service lines to be a danger in terms of lead exposure. In Table S10, we show that our results are robust to alternative definitions, such as only considering lead lines to be dangerous and copper service lines to be not dangerous. In addition to information on service line materials, the inspection data includes a host of other variables describing the housing unit, including: the use type (residential, commercial, industrial), rental or owner-occupied, year built, the condition of the house unit in 2014 (good, fair, poor, or structurally deficient), assessed building value, and assessed land value.

Past research utilized the same service line inspections data, combined with home water testing results, to show that service lines were a key source of lead exposure in Flint. During the peak of the crisis, children living in homes with lead service lines consumed 4.5 times the amount of lead per day than children residing in homes with copper pipes [15] (galvanized steel service lines, which we include in our primary "lead" treatment definition, were associated with 1.8 times more lead per day than copper pipes). However, by 2017, children in homes with lead service lines were no longer consuming a meaningful amount of lead in their home drinking water. By matching data on residential water tests with data on pipe material for a subset of students (see Section A4 of the appendix), we were able to confirm that similar patterns applied in our analytic sample; in our student sample, homes with lead or galvanized steel service lines

were 4.7 percentage points (P<.01), or 77%, more likely to exhibit water lead levels above the EPA threshold of 15 ppb.

To create our within-Flint analytic sample, we start with the 17,024 students who were living in Flint and enrolled in Michigan public schools during the 2013-2014 school year. Note, that our analytic sample here is slightly larger than the sample used in our synthetic control analysis. There, we focus only on grades K-12 because districts across the state may vary in their pre-K enrollment and high school matriculation policies. In our within-Flint analysis, where our identification strategy relies on within-student comparisons over time, we elect to include observations of students in pre-K and in ungraded classrooms.

Using a probabilistic matching algorithm based on street number, street direction, street name, and street type, we were able to identify the service line material for 10,245 students, or 60.18% of the initial sample. This address-level match was executed on our behalf by staff at the University of Michigan, who obtained special permission from the Michigan Department of Education to use personally identifiable information. Students' home addresses, as well as all other personally identifiable information, were stripped from the matched data before it was returned to our research team. A small fraction of the non-matches, roughly 1%, were due to missing or invalid address information in either the pipes data or the student education records. A manual review conducted on a random subsample of the data suggests that our matching algorithm failed to match roughly 18% of true matches. Thus, the primary reason for unmatched students is likely the limited coverage of the pipes data.

While the match rate will not affect the internal validity of our estimates, it may limit the generalizability of our findings. To help understand how well results from the matched sample may generalize to the full population of Flint students, Table S5 compares the full set of Flint students to the set whose addresses matched the pipes data. The matched students appear extremely similar to the unmatched students, particularly when we compare students within the same census blocks (Column 5), which suggests that our results should generalize well to the broader population of Flint children.

Water Test Data

For certain auxiliary analyses, we use data on residential water test results obtained from the state of Michigan (we refer to this data as "water data") at the following link: https://www.michigan.gov/flintwater/0,6092,7-345-76292_76294_76297---,00.html. The water data contains results from lead tests using a voluntary homeowner-driven sampling program whereby concerned citizens were provided testing kits and conduct sampling on their own. We use data from water tests conducted in 2016, which consists of 17,421 addresses in the city of Flint. Because many addresses had multiple tests within 2016, we create a single binary variable that indicates whether the address had at least one water test return a value of lead concentration greater than one part per billion.

Next, we matched our household water testing data to our analytic sample of 17,024 students to any of the 17,421 unique addresses with valid water test data in 2016. To do so, University of Michigan research staff first parsed addresses to facilitate the algorithm responsible for the match using the ArcGIS Geocoding Parser. Parsing involved splitting an address into various fields (e.g., address number, street name pre-directional such as north or south, street name, zip code). This step was not necessary for the service line materials addresses because they had been previously parsed using the Google Maps API by the researchers who collected the data. From there, they attempted to match students to water test results from the water data

using student home address. Home water test results were matched to 5,355 students, or 31.46% of our analytic sample 17,024. Both pipes and water data were matched to just 3,430 students. The comparatively fewer Flint addresses with water data largely explains why we were able to match a smaller proportion of students to water test results.

Determinants of Lead Pipes

Using the language of program evaluation, our within-Flint analysis considers children living in housing units with lead pipes in 2014 as the treatment group and their peers living in housing units with copper pipes as the control group. If children were randomly distributed across housing units in Flint, one could estimate the impact of lead exposure simply by comparing outcomes of children living in homes with and without lead pipes. However, given Flint's historical growth patterns and the decreases over time in the installation of lead pipes, there is reason to believe that homes in certain neighbourhoods are more likely to have lead piping than others; an 1897 Flint city ordinance required "all connections with any water mains be made with lead pipe" [52], and the use of lead pipes was slowly phased out over the 21st century.

To examine the determinants of lead pipes in our analytic sample, Table S6 presents results from several regression models that include both household-level and neighborhood-level predictors. Column 1 shows that a small set of house characteristics are strong predictors of the presence of lead piping. For example, older houses are more likely to contain lead pipes; all else equal, a house that is 10 years older is 20 percentage points more likely have lead pipes, a large effect given that just 40% of all houses have lead pipes. Conditional on age, rental status, and house condition, a $10,000 increase in the assessed home value is associated with a 0.6 percentage point reduction in the likelihood of lead service lines.

Column 2 shows that, conditional on house characteristics, units located in higher poverty census blocks are more likely to have lead piping, although the magnitude of this relationship is modest; a 10 percentage point increase in the poverty rate is associated with a 2.5 percentage point increase in the likelihood of lead piping. Interestingly, the magnitude of the coefficient on the neighborhood poverty variable does not change much if one omits the other census block characteristics.

When we include census block fixed effects in Column 3, we find that approximately 38% of the variation in the presence of lead pipes occurs between (as opposed to within) census blocks, but housing unit characteristics explain only about a third of this within-block variation. Put differently, there is considerable variation in the presence of dangerous piping even among observationally identical housing units located in the same census block, consistent with a quasi-random distribution of pipe materials across houses of similar vintage in similar neighborhoods (indeed, the city and homeowners alike largely did not know which houses had lead services lines prior to the FWC). This fact forms the basis for our within-Flint empirical strategy.

Difference-in-Differences Estimation

We next examine the relationship between pipe material and student and school characteristics. Table S7 compares the characteristics of students living in homes with and without lead pipes. Students in homes with lead pipes are more likely to be Black (7.9 percentage points) and economically disadvantaged (3.1 percentage points). Columns 5 and 6 present the difference across groups from regressions that includes census block fixed effects. Additionally, the specification reported in Column 6 controls for the house characteristics listed in Table S6.

Once we condition on neighborhood and house characteristics, the small to modest differences between students living in homes with lead versus copper pipes diminish substantially. Indeed, none of the 11 student characteristics in the top panel are statistically significant at the 5% level in Columns 5 or 6. Across 13 individual demographic characteristics and measures of academic performance, there are only 3 differences that are statistically significant at the 5% level: school location (city versus suburb), magnet school, and school racial composition.

In summary, children living in homes that were most susceptible to lead exposure were observationally similar to children in homes with copper pipes that presented no lead danger. Nonetheless it is possible that the two groups of children differ in unobservable ways that influence their educational outcomes after the water crisis. To account for unobservable time-invariant child and family characteristics, we estimate difference-in-differences (DiD) models.

To this end, we construct a student-year panel for the 10,245 matched students that runs from the 2009-10 through 2018-19 academic years. This allows us to examine up to five years pre and five years post the FWC. Note that this is an unbalanced panel because not all children appear in all years (e.g., many of the older children in 2014 will have graduated or dropped out prior to 2019, and the very young children in 2014 were not yet enrolled in public school in 2010).

In order to produce DiD estimates of the average treatment effect of having lead pipes during the Flint Water Crisis, it is standard to estimate a two-way fixed effects model for outcome y for student i in year t:

$$y_{it} = \lambda_i + \delta_t + P_t^{t>2014} D_i + \varepsilon_{it}$$

(S8)

λ_i: Student fixed effects
δ_t: Year fixed effects
$P_t^{t>2014}$: Post-treatment period dummy variable
D_i: Lead service line dummy variable

The identifying assumption of the DiD model is commonly referred to as "parallel trends," meaning that in the absence of the exposure, the outcomes of the treatment group would follow the same path as that of the control group. A standard approach to testing the plausibility of this assumption is to estimate an event study model, which is simply an extension of the two-way fixed effects model that allows the effect of the exposure to differ by year relative to the start of the treatment. This corresponds to the following regression model:

$$y_{it} = \lambda_i + \delta_t + \sum_{T=2011}^{2014} \gamma_t^{t=T} D_i + \sum_{T=2015}^{2019} \gamma_t^{t=T} D_i + \varepsilon_{itk}$$

(S9)

$\gamma_t^{t=T}$: Year dummy variable

The coefficients γ_t trace out the differences by year in outcomes between students living in homes with and without lead service lines, with 2010 serving as the omitted year. The estimates of γ_t from 2011 through 2014 serve as a specification check, whereas the estimates from 2015 to 2019 reflect the impact of greater exposure to lead.

While differences-in-differences has become a popular strategy for estimating causal effects of in non-experimental settings, a growing literature highlights several important

limitations of the standard two-way fixed effects approach [78]–[80]. These papers point out that the canonical regression DiD estimate relies on the strong assumption of treatment effect homogeneity. For this reason, we utilize the novel imputation estimator proposed by Borusyak, Jaravel, and Spiess 2021 [79], which has the dual advantages of being intuitively clear and straightforward to implement.

We first estimate the two-way fixed effects model described above, using only the untreated observations, which includes all observations of the control students (i.e., those living in homes without lead pipes) as well as the pre-treatment observations for treatment students (i.e., years 2010 through 2014). Using these estimates, we generate predicted (imputed) values of the outcome for treatment students in the post-treatment period. For each student-year observation, we then calculate the difference between their observed and predicted outcome (which each correspond to students' potential outcome in the treated state and untreated state, respectively). These student-year effects can then be aggregated in various ways. To recover the canonical difference-in-differences estimate, we calculate a simple (unweighted) average of all treatment group students in all post-treatment years. To estimate the year-specific "event study" effects shown in equation 6.ii.b, we generate separate averages for each year. While visual inspection of these pre-exposure estimates can be useful, we also conduct formal tests of the null hypothesis that the pre-exposure effects are jointly significant. Standard errors that account for heterogeneity and serial correlation within students over time are calculated via a bootstrap procedure outlined in Borusyak, Jaravel, and Spiess 2021 [79]. We implement this procedure using the Stata command `did_imputation`.

In our baseline models, we only include student and year fixed effects. To increase our statistical power and test the sensitivity of our results, we estimate additional models that include a variety of added controls. We first add school-grade fixed effects to limit our comparison to students in comparable educational settings. We then add race-gender-year fixed effects, interactions between a student's grade in 2013-2014 and year dummies, and interactions between census block poverty in 2013-2014 and year dummies.

One final concern involves differential attrition. If the water crisis caused Flint students living in homes with lead pipes to exit the public school system differently than other Flint students, our DiD estimates could be biased. To determine whether students living in homes with lead pipes in 2014 were more likely to leave the Michigan public schools, we estimated a series of OLS regression models. The coefficient on lead pipes was consistently very small and not close to statistical significance, regardless of the control variables included (including none) or the year examined. This suggests that attrition bias is not a concern in the results discussed below.

Results

Figure 6 displays connected scatterplots of our four main academic outcomes, with the trends broken out separately by home service line material. In it, we see only small differences between the trends of students with lead pipes and students with copper pipes before and after the crisis. These descriptive patterns foreshadow the largely small or null within-Flint results in our DiD and event study models.

The results from our event study models are displayed in Figure S4. Beginning with our baseline two-way fixed effect model in the left panel and focusing on the coefficients prior to treatment in 2014, we see little evidence for differential trends across students with and without lead pipes. In three of the four outcomes, a test of the joint significance of the pre-exposure

effects is not significant. In the case of math achievement, however, we do reject the null hypothesis that treatment student trends were equal to those of control students. While there is not a clear pattern to the pre-exposure trend, math achievement appears to dip in the final pretreatment year.

As described earlier, because students were not randomly assigned to homes with lead pipes, it is likely that there may be some differences between our treatment and control groups. To account for some of the observable differences, we re-estimate our event study models including the large set of controls described above (shown in the right panel of Figure S4). While the joint test of pre-trends for math is no longer statistically significant (p=0.14), a similar observational pattern emerges. However, because we would a priori expect negative effects of dangerous pipes on math achievement, this potential violation merely entails that our math affects are biased to be *larger* in magnitude. When viewing these figures, it is important to keep in mind that the composition of the treatment and control groups are changing across time periods as students graduate, drop out, or (for academic achievement) move in/out of grades 3-8.

Results from classic OLS DiD models are displayed in Table S8. Results from DiD imputation models are displayed in Table S9 and are highly comparable to classic model results. In the baseline two-way fixed effects models, dangerous services lines have a -0.03 SD effect on math achievement, a -0.02 SD effect on reading achievement, a 0.5 percentage point effect on special needs status, and a 0.02 percentage point effect on daily attendance. In our most saturated models, with school-grade fixed effects and a variety of other controls, dangerous service lines have a -0.02 effect on math achievement, a -0.01 effect on reading achievement, a 0.26 percentage point effect on special needs status, and a 0.07 percentage point effect on daily attendance. None of the coefficients on our four educational outcomes are statistically significant in any of the four specifications.

In general, the small, statistically insignificant effects that we observe within-Flint stand in stark contrast to the dramatic decline in math achievement and increase in special needs status that we observe for all Flint students in our between-district synthetic control analysis. Importantly, our DiD analyses are not powered to detect small effects. For example, using a 95% confidence interval around the estimates from our most saturated model, we can only rule out an increase special needs status greater than in 0.60 percentage points and decreases greater than 0.060 SD for math achievement, 0.063 SD for reading achievement, and 0.25 percentage points for attendance.

Our DiD results are robust to various alternative specifications that use different definitions of lead and copper service lines (see Table S10). Specifically, one might be concerned that our results may be sensitive to the way in which we defined various service line materials connoting lead risk. To address this concern, we construct two additional definitions for lead service lines and copper service lines, each more restrictive than the last; our results are not sensitive to differences in how we define lead treatment.

SUPPLEMENTARY TABLES & FIGURES

Figure S1. Observations of Educational Outcomes Over Time in Flint.

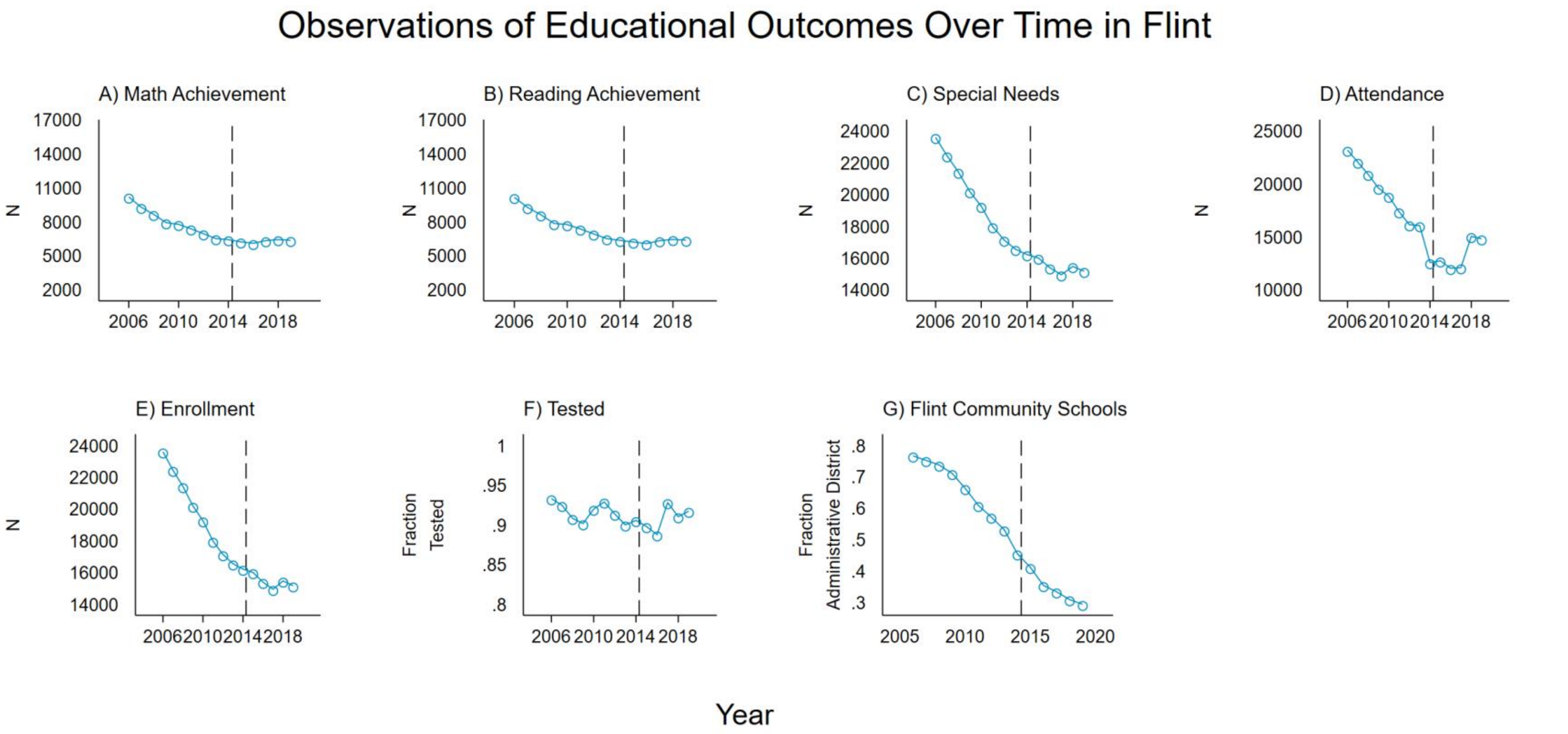

Note: This figure displays descriptive trends in the number of observations of academic outcomes and trends in selected student enrollment characteristics for the Flint geographic district from 2006-2019. Data is taken from the Michigan Department of Education's longitudinal administrative data base. The grey dotted vertical line represents time that the Flint Water Crisis begins. Math and reading achievement are observed in only grades 3-8, whereas special needs and attendance are observed in grades K-12. Traditional sample is used for our main synthetic control analysis, while the fixed sample is use for a robustness check described in Section 5C.

Figure S2. Visualizing Within-Michigan and Out-of-Sample Attrition.

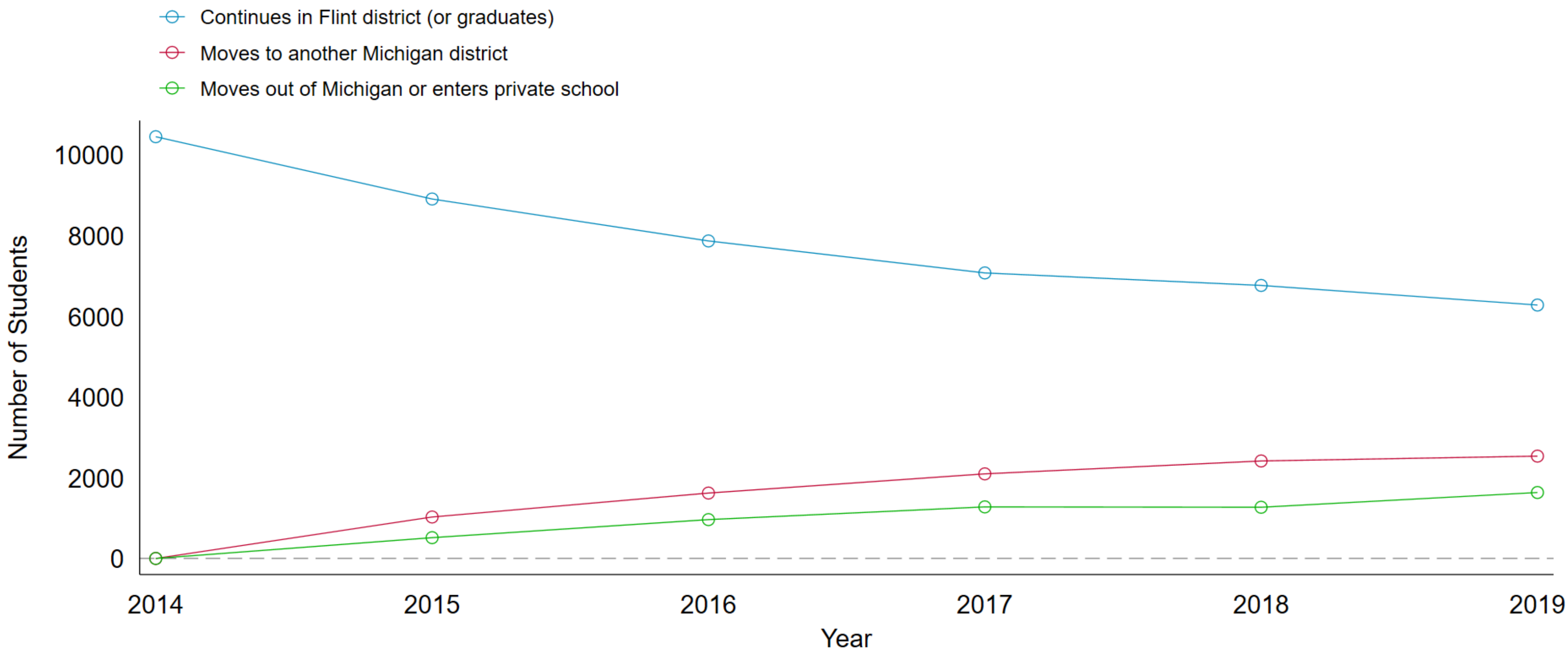

This figure follows 10,450 Flint students who were in K-7th grade during 2013-2014 schools year. Attrition to another school district in Michigan, which can be accounted for by our invariant district assignment strategy, displayed in red. Attrition to private school or a public school outside of Michigan is display in green.

Figure S3. Synthetic Control Results for Chronic Absenteeism.

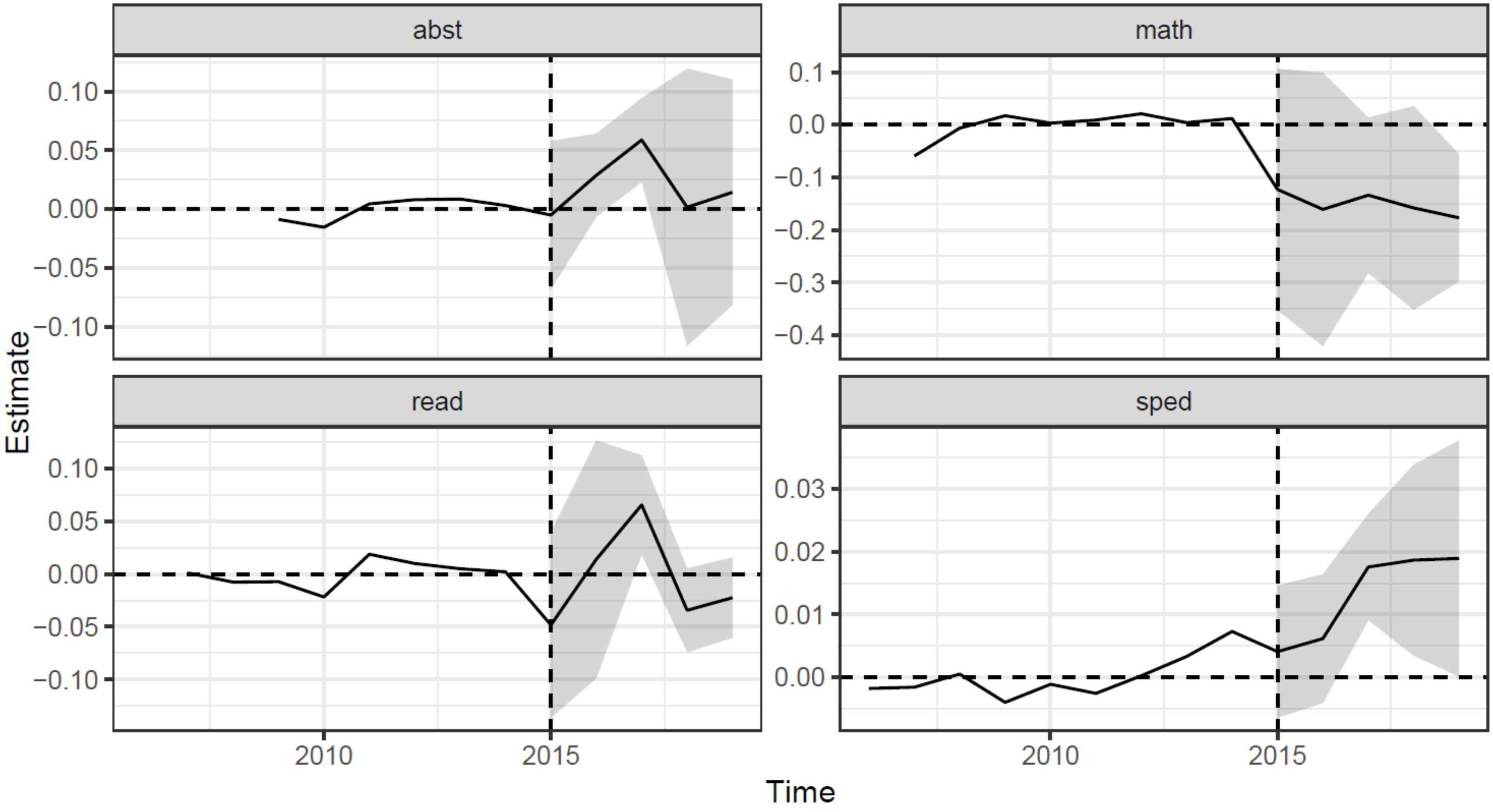

This figure displays synthetic control results. The model is identical to our primary specification, except the outcome of daily attendance is swapped with chronic absenteeism ("abst"). Chronic absenteeism is defined as the fraction of students who are absent more than 10% of days in a given school year. The average treatment effect for chronic absenteeism is 1.97 (2.37) percentage points and is statistically insignificant.

Figure S4. Event Study Imputation Estimates of Lead Pipes on Student Outcomes.

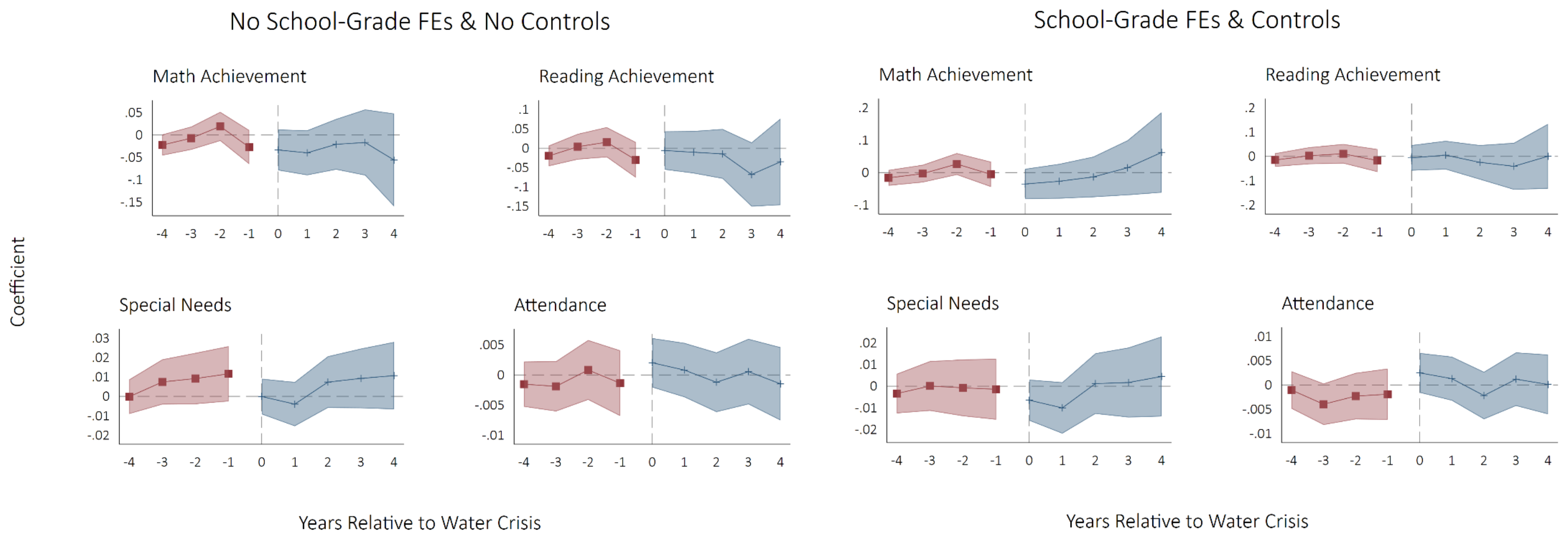

Note. This figure displays Dangerous Pipes * Year Dummy event study coefficients. Confidence intervals (95%) are shown in shaded red and blue areas. Lead Pipes * 2010, the first year in our Flint student-level panel, is the omitted category. The left four figures correspond to Models 1, 5, 9, and 13 and the right four figures correspond to models 4, 8, 12, and 16 from Table S9, respectively. Controls include race-gender-year fixed effects, a vector of interactions between a student's grade in 2013-2014 and year dummies, and a vector of interactions between a student's census block poverty in 2013-2014 and year dummies.

Table S1. Prior Estimates of Lead on Achievement.

**Aizer et al. 2018 [*21*]**	Blood Lead IV		Certificate IV	
	Test Score	Test Score/SD	Test Score	Test Score/SD
Reading (Age 8)	-0.396	-0.030	-0.931	-0.072
	[0.0372]	[0.003]	[0.516]	[0.040]
Math (Age 8)	-0.266	-0.020	-0.431	-0.033
	[0.0347]	[0.003]	[0.471]	[0.036]

**Rueben et al. 2017 [*10*]**	Unadjusted		Covariate Adjusted*	
	Test Score	Test Score/SD	Test Score	Test Score/SD
Cig (Age 38)	-0.394	-0.026	-0.322	-0.021
	[0.140]	[0.009]	[0.089]	[0.006]

*Covariates include sex, maternal IQ, childhood IQ (age 11), and childhood socioeconomic status.

**Lanphear et al. 2005 [*74*]**	Covariate Adjusted**	
	Test Score	Test Score/SD
IQ (Age 7)	-0.620	-0.032
	[0.133]	[0.007]

**Covariates include birth weight, maternal IQ, and maternal education.

Note: This table displays previous estimates of the effect lead on achievement taken from three studies: Aizer et al. 2018 [*21*], Reuben et al. 2017 [*10*], and Lanphear et al. 2005 [*74*].

Table S2. List of 54 Control Districts.

District Code	District Name
13020	Battle Creek Public Schools
82160	Wayne-Westland Community School District
80020	Bangor Public Schools (Van Buren)
25240	Beecher Community School District
25060	Bendle Public Schools
11010	Benton Harbor Area Schools
80090	Bloomingdale Public School District
73180	Bridgeport-Spaulding Community School District
25080	Carman-Ainsworth Community Schools
50070	Clintondale Community Schools
82030	Dearborn City School District
82040	Dearborn Heights School District #7
82240	Westwood Community School District
82010	Detroit City School District
14020	Dowagiac Union School District
50020	East Detroit Public Schools
82250	Ecorse Public Schools
63020	Ferndale Public Schools
50090	Fitzgerald Public Schools
41120	Godfrey-Lee Public Schools
41020	Godwin Heights Public Schools
41010	Grand Rapids Public Schools
82060	Hamtramck School District
82320	Harper Woods The School District
18060	Harrison Community Schools
80120	Hartford Public Schools
63130	Hazel Park School District
72020	Houghton Lake Community Schools
38170	Jackson Public Schools
39010	Kalamazoo Public Schools
41140	Kelloggsville Public Schools
41160	Kentwood Public Schools
33020	Lansing Public School District
81070	Lincoln Consolidated School District
82090	Lincoln Park School District
82045	Melvindale-North Allen Park Schools
50160	Mount Clemens Community School District
25040	Mt. Morris Consolidated Schools
61010	Muskegon Public Schools
63250	Oak Park School District
61190	Orchard View Schools
63030	Pontiac City School District
82110	Redford Union Schools District No. 1
82120	River Rouge School District
82130	Romulus Community Schools
73010	Saginaw School District
50200	South Lake Schools
82140	South Redford School District
63060	Southfield Public School District
82430	Van Buren Public Schools
50220	Van Dyke Public Schools
33215	Waverly Community Schools
35040	Whittemore-Prescott Area Schools
81020	Ypsilanti Community Schools

Note. The control samples were selected from the distribution of all Michigan geographic districts with enrollment greater than 1,000 students during the 2013-2014 academic year (See Panel A of Figure 3). Data is taken from the Michigan Department of Education’s longitudinal administrative data base.

Table S3. Descriptive Statistics: Time Invariant District Assignment Panel.

	2010		2014	2018	
Traditional Sample	X		X	X	
Invariant District Assignment Sample		X	X		X
Math Achievement	-0.58	-0.59	-0.51	-0.73	-0.73
Reading Achievement	-0.62	-0.64	-0.53	-0.68	-0.68
Fraction Special Needs	0.14	0.16	0.15	0.16	0.18
Fraction School Days Attended	0.92	0.93	0.91	0.89	0.89
Fraction Female	0.49	0.49	0.49	0.48	0.48
Fraction Black	0.77	0.78	0.76	0.69	0.69
Fraction Hispanic	0.04	0.04	0.04	0.05	0.05
Fraction Economically Disadvantaged	0.77	0.87	0.89	0.92	0.90
Fraction Limited English Proficiency	0.02	0.02	0.03	0.04	0.04
Fraction Attending Charter Schools	0.25	0.27	0.31	0.37	0.27
Fraction Attending Administrative District	0.66	0.54	0.45	0.31	0.25
Number of Observations	19,254	10,141	16,210	15,466	11,213

Note. This table contains geographic school district characteristics from the Michigan Department of Education's longitudinal administrative data. Math and reading achievement are standardized within test subject, grade, and year to the overall state distribution scores. Math and reading achievement are observed in only grades 3-8, whereas all other variables as observed in grades K-12. The Traditional Sample defines a student's geographic district using a student's home address *in that year*, whereas the Invariant District Assignment Sample defines a student's geographic district using a student's home address during the 2013-2014 school year (final pre-treatment period). Thus, the Traditional and Invariant District Assignment samples for 2013-2014 are mechanically identical.

Table S4. Synthetic Control Robustness: Weights & Selected District Covariates.

	Synthetic Control Weights				***District Covariates***		
**District ID**	**Original**	**Invariant District Assignment**	**Alternative Controls I (∩75)**	**Alternative Controls II (∪95)**	**Enrollment**	**Fraction Black**	**Fraction Economically Disadvantaged**
63250	0.19	0.06	0.20	0.20	3902	0.90	0.70
81070	0.15	0.09	—	—	5835	0.32	0.43
14020	0.15	0.00	0.11	—	2595	0.15	0.75
82060	0.13	0.11	0.11	0.17	5007	0.20	0.95
82120	0.10	0.00	0.15	0.16	1491	0.64	0.91
72020	0.09	0.00	—	—	1620	0.02	0.74
35040	0.08	0.00	—	0.00	1021	0.02	0.88
82430	0.05	0.00	—	—	6826	0.43	0.49
25240	0.04	0.28	0.07	0.20	1736	0.88	0.90
80090	0.01	0.00	—	0.08	1534	0.06	0.81
25080	0.00	0.00	0.08	—	4625	0.49	0.72
33020	0.00	0.00	0.02	—	17707	0.43	0.73
63030	0.00	0.00	0.00	0.02	11184	0.59	0.80
41160	0.00	0.00	0.02	—	10746	0.33	0.64
25040	0.00	0.00	0.00	0.06	2423	0.20	0.82
11010	0.00	0.11	0.00	0.00	6009	0.76	0.87
81020	0.00	0.00	0.00	0.02	6810	0.57	0.68
61010	0.00	0.16	0.02	0.09	6165	0.54	0.87
33215	0.00	0.17	—	—	2670	0.34	0.53
82150	—	—	0.18	—	10643	0.31	0.72
63140	—	—	0.03	—	1724	0.18	0.69
Flint	—	—	—	—	16210	0.76	0.89

Note. This table displays weights from four synthetic control models using a sample of geographic school districts taken from the Michigan Department of Education’s longitudinal administrative data base.

Table S5. Student Education Record to Home Service Line Data Match.

		Flint Students Matched to Service Line Data			
	All Flint students (n=17,024)	Matched (n = 10,245)	Not Matched (n = 6,779)	Difference	Difference (w/census block fixed effects)
	(1)	(2)	(3)	(4)	(5)
**Student**					
Math Achievement (z-score)	-0.51	-0.50	-0.52	0.023	-0.014
Reading Achievement (z-score)	-0.53	-0.51	-0.57	0.052*	0.031
Special Needs (%)	0.16	0.16	0.15	0.014*	0.022*
School Days Attended (%)	0.91	0.91	0.90	0.010**	0.009**
Female (%)	0.49	0.48	0.49	-0.006	0.003
Black (%)	0.76	0.74	0.79	-0.047**	-0.006
Hispanic (%)	0.04	0.05	0.03	0.012**	0.006
Economically Disadvantaged (%)	0.88	0.87	0.90	-0.030**	-0.025**
Limited English Proficiency (%)	0.03	0.03	0.02	0.017**	0.009*
Flint Community Schools (%)	0.45	0.48	0.41	0.061**	0.053**
**School**					
City (%)	0.57	0.59	0.54	0.055**	0.056**
Suburb (%)	0.38	0.36	0.42	-0.061**	-0.066**
Charter (%)	0.30	0.27	0.35	-0.085**	-0.076**
Magnet (%)	0.54	0.58	0.50	0.080**	0.068**
Enrollment (N)	598.41	600.33	595.51	3.902	-24.590*
Economically Disadvantaged (%)	0.80	0.80	0.81	-0.016**	-0.017**
Black (%)	0.67	0.66	0.68	-0.019*	-0.018**
Hispanic (%)	0.04	0.04	0.04	0.002*	0.003**
White (%)	0.24	0.25	0.23	0.015*	0.014*
First Year Teachers (%)	0.05	0.05	0.05	-0.003	-0.001
District per-pupil expenditures ($)	6410.22	6567.91	6172.36	379.687**	307.118**
**Neighborhood**					
Black (%)	0.57	0.56	0.60	-0.036**	
Hispanic (%)	0.03	0.03	0.04	-0.003	
Age 65+ (%)	0.12	0.12	0.11	0.010**	
Age 25+: BA (%)	0.10	0.11	0.10	0.009	
Age 25+: <HS Degree (%)	0.18	0.17	0.18	-0.014**	
Below Poverty Line (%)	0.42	0.40	0.45	-0.052**	
Unemployed (%)	0.28	0.27	0.29	-0.012	
Owner-occupied Houses (%)	0.43	0.46	0.39	0.067**	

Note. This table compares the full set of Flint students to the set whose addresses matched the service line data. To estimate the differences between the matched and unmatched sample, we used a series of regressions that adjusted the standard errors for clustering at the school-level and census block. For the student-level characteristics, we clustered the standard errors at the school level for all variables except percent attending Flint, another school district, charter, and living in Flint in 2013-2014, for which we adjusted standard errors using census block. For school-level variables, we used adjusted standard errors for census block.

Table S6. The Determinants of Lead Pipes in the Homes of Flint Children.

	(1)	(2)	(3)
**Housing characteristics**			
Year built	-0.020***	-0.019***	-0.016***
	(0.001)	(0.001)	(0.000)
Housing condition 2014: Poor	0.050*	0.045*	0.002
	(0.026)	(0.026)	(0.015)
Housing condition 2014: Fair	-0.008	-0.015	-0.008
	(0.017)	(0.017)	(0.008)
Residential value ($10,000)	0.006**	0.007**	0.006***
	(0.003)	(0.003)	(0.001)
Land improvement flag	-0.033*	-0.019	-0.017
	(0.020)	(0.019)	(0.011)
Rental	0.004	-0.000	0.005
	(0.012)	(0.012)	(0.007)
Missing: Year built	-38.114***	-36.826***	-30.510***
	(1.477)	(1.559)	(0.475)
**Neighborhood characteristics**			
% Black		-0.001*	
		(0.000)	
% Hispanic		-0.003	
		(0.002)	
% Persons: 65 and over		0.001	
		(0.002)	
% Persons 25 years and over with bachelors		0.002	
		(0.001)	
% Persons 25 years and over with less than HS		-0.000	
		(0.001)	
% Economically Disadvantaged		0.002**	
		(0.001)	
% Unemployed		0.001	
		(0.001)	
% Owner-occupied		-0.002**	
		(0.001)	
Constant	38.817***	37.495***	31.132***
	(1.476)	(1.563)	(0.477)
Fixed effects for census blocks?	No	No	Yes

Notes: N=10,180
*p < 0.05, **p < 0.01, ***p < 0.001

Note. This table presents results from several regression models that include both household-level and neighborhood-level predictors.

Table S7. Comparing Flint Children with Lead versus Copper Pipes.

	All matched students (n = 10,245)	Copper Pipes (*n* = 6,183)	Lead Pipes (*n* = 4,062)	Difference	Difference (w/census block fixed effects)	Difference (w/ census block fixed effects and housing characteristics)
	(1)	(2)	(3)	(4)	(5)	(6)
**Student**						
Math Achievement (z-score)	-0.5	-0.51	-0.49	0.013	-0.029	-0.025
Reading Achievement (z-score)	-0.51	-0.53	-0.5	0.029	-0.034	-0.035
Special Needs (%)	0.16	0.16	0.17	0.007	0.009	0.01
School Days Attended (%)	0.91	0.92	0.91	-0.005	0.003	0.001
Female (%)	0.48	0.48	0.49	0.002	0.001	-0.033
Black (%)	0.74	0.77	0.69	-0.079**	-0.012	0.022
Hispanic (%)	0.05	0.04	0.06	0.017*	0.001	-0.002
Economically Disadvantaged (%)	0.87	0.86	0.89	0.031**	0.025	0.022
Limited English Proficiency (%)	0.03	0.03	0.05	0.019**	0.01	0.011
Attending school in Flint (%)	0.48	0.45	0.51	0.058**	0.039	0.044
Attending another school district (%)	0.25	0.27	0.23	-0.035**	-0.033	-0.038
**School**						
City (%)	0.59	0.56	0.63	0.072**	0.044	0.053*
Suburb (%)	0.36	0.38	0.32	-0.055**	-0.043	-0.051*
Charter (%)	0.27	0.28	0.25	-0.024*	-0.006	-0.007
Magnet (%)	0.58	0.56	0.6	0.035**	0.04	0.059*
Enrollment (N)	600.33	606.51	590.94	-15.746	-11.637	-3.789
Economically Disadvantaged (%)	0.8	0.79	0.8	0.008*	0.012*	0.011
Black (%)	0.66	0.66	0.66	0.002	0.017	0.028*
Hispanic (%)	0.04	0.04	0.04	0.002*	-0.001	-0.001
White (%)	0.25	0.25	0.25	-0.003	-0.015	-0.026*
FTE teachers in their first year of teaching (%)	0.05	0.05	0.04	-0.002	-0.002	-0.003
Per-pupil district-level total instructional expenditures ($)	6567.91	6492.08	6683.42	190.476**	75.048	65.572
**Neighborhood**						
Black (%)	0.56	0.6	0.5	-0.094**		
Hispanic (%)	0.03	0.03	0.04	0.005*		
Age 65 and over (%)	0.12	0.13	0.11	-0.020**		
Age 25 and over: BA (%)	0.11	0.11	0.1	-0.011*		
Age 25 and over: less than high school (%)	0.17	0.16	0.19	0.024**		
Economically Disadvantaged (%)	0.4	0.37	0.44	0.070**		
Unemployed (%)	0.27	0.27	0.29	0.019**		
Owner-occupied Houses (%)	0.46	0.48	0.42	-0.068**		

Note. $*p < 0.05$, $**p < 0.01$

Service line material is considered dangerous based on pipes made out of lead. Water is coded as dangerous if it contains >15 PPB of lead.
[a]The difference column was calculated by regressing dangerous status on each of the variables listed on the table with census blocks fixed effects.
For the service line materials, the overall F-test using all listed variables to predict dangerous status was significant, $F(12, 1663) = 1.49$, $p = 0.12$.
For the water material, the overall F-test using all listed variables to predict dangerous status was significant, $F(10, 1534) = 0.96$, $p = 0.487$. Education variables are taken from 2013-2014, the final year pretreatment.

Table S8. Traditional Difference-in-Differences Results: Lead Service Line.

	Math Achievement		Reading Achievement		Special Needs		Attendance	
	(1)	(2)	(3)	(4)	(5)	(6)	(7)	(8)
Lead Pipes * Post	-0.0387	-0.0262	-0.00875	-0.00168	-0.00537	-0.00382	-0.00369*	-0.000536
	(0.0199)	(0.0191)	(0.0225)	(0.0220)	(0.00540)	(0.00526)	(0.00174)	(0.00160)
Control Mean	{-.62}	{-.62}	{-.60}	{-.60}	{.176}	{.175}	{.917}	{.917}
School-Grade Fixed Effect		X		X		X		X
Students	8219	8094	8208	8073	10180	10165	10001	9931
Student-Years	35933	35104	35837	34992	80753	79158	67438	66118

**p* < 0.05, ***p* < 0.01

Note. This table displays results from OLS difference-in-differences regressions of the effect of having dangerous pipe materials on a student's academic outcomes during the Flint Water Crisis using a panel spanning 2010-2019. All models include student fixed effects and year fixed effects. Education data is taken from the Michigan Department of Education's longitudinal administrative data base. Service line material data was collected during the City of Flint's service line inspection and replacement program that was implemented in the aftermath the crisis. Lead or galvanized steel service lines are classified as dangerous, whereas copper service lines are classified as not dangerous. These difference-in-differences models are estimated using OLS regression.

Table S9. Difference-in-Differences Imputation Results: Lead Service Line.

	Math Achievement				Reading Achievement			
	(1)	(2)	(3)	(4)	(5)	(6)	(7)	(8)
Lead Pipes * Post	-0.0319	-0.0314	-0.0198	-0.0161	-0.0190	-0.0152	-0.0199	-0.0110
	(0.0218)	(0.0233)	(0.0212)	(0.0225)	(0.0250)	(0.0267)	(0.0246)	(0.0264)
Control Mean	{-.62}	{-.62}	{-.62}	{-.62}	{-.60}	{-.60}	{-.60}	{-.60}
Controls		X		X		X		X
School-Grade Fixed Effects			X	X			X	X
Pre-Trends P-Value	0.0219	0.107	0.0504	0.137	0.122	0.473	0.165	0.526
Students	8034	8018	7959	7944	8024	8008	7953	7938
Student-Years	33159	33084	32851	32781	33057	32982	32755	32685
	Special Needs				Attendance			
	(9)	(10)	(11)	(12)	(13)	(14)	(15)	(16)
Lead Pipes * Post	0.00398	-0.00553	-0.000318	-0.00255	0.000225	-0.00269	-0.000740	0.000700
	(0.00558)	(0.00587)	(0.00559)	(0.00581)	(0.00169)	(0.00172)	(0.00159)	(0.00166)
Control Mean	{.176}	{.176}	{.175}	{.174}	{.916}	{.917}	{.916}	{.916}
Controls		X		X		X		X
School-Grade Fixed Effects			X	X			X	X
Pre-Trends P-Value	0.365	0.753	0.862	0.796	0.640	0.412	0.183	0.433
Students	10245	10225	10238	10218	9921	9898	9866	9847
Student-Years	80818	80654	79642	79477	66797	66666	66208	66092

$*p < 0.05$, $**p < 0.01$

Note. This table displays results from difference-in-differences regressions of the effect of having dangerous pipe materials on a student's academic outcomes during the FWC using a panel spanning 2010-2019. All models include student fixed effects and year fixed effects. Education data is taken from the Michigan Department of Education's longitudinal administrative data base. Service line material data was collected during the City of Flint's service line inspection and replacement program that was implemented in the aftermath the crisis. Models with controls include race-gender-year fixed effects, a vector of interactions between a student's grade in 2013-2014 and year dummies, and a vector of interactions between a student's census block poverty in 2013-2014 and year dummies. These difference-in-differences models are estimated using imputation (Borusyak, Jaravel, and Spiess 2021).

Table S10. Difference-in-Differences Results: Alternative Lead/Copper Definitions.

	(1)	(2)	(3)	(4)
	Math Achievement	Reading Achievement	Special Needs	Attendance
A.				
Lead Pipes * Post	-0.0262	-0.00168	-0.00382	-0.000536
	(0.0191)	(0.0220)	(0.00526)	(0.00160)
Control Mean	{-.62}	{-.60}	{.175}	{.917}
Students	8094	8073	10165	9931
Student-Years	35104	34992	79158	66118
B.				
Lead2 Pipes * Post	-0.0253	0.00351	-0.00479	-0.00145
	(0.0194)	(0.0223)	(0.00533)	(0.00164)
Control Mean	{-.62}	{-.60}	{.175}	{.917}
Students	7907	7886	9913	9683
Student-Years	34290	34183	77217	64475
C.				
Lead3 Pipes * Post	-0.0292	-0.00288	-0.00427	-0.00167
	(0.0197)	(0.0225)	(0.00540)	(0.00166)
Control Mean	{-.62}	{-.60}	{.176}	{.917}
Students	7723	7702	9685	9460
Student-Years	33504	33399	75436	63008

* $p<.05$; ** $p<.01$

Note. This table displays results from difference-in-differences regressions of the effect of having lead + copper (as opposed to only lead) pipe materials on a student's academic outcomes during the Flint Water Crisis using a panel spanning 2010-2019. All models include student fixed effects and year fixed effects and control for having galvanized steel pipes. In the difference-in-differences models presented in the main text, the "Lead" variable is equal to l for all service line materials considered dangerous (and scheduled for replacement by Flint's FAST Start team) and is equal to 0 for all other service line materials. In the Panel A above, the "Lead2" variable is equal to 1 only for lead or galvanized steel service, equal to 0 for only copper or safe non-copper service lines, and is set to missing otherwise. In Panel B above, the "Lead3" variable is equal to 1 for only lead service lines, is equal to 0 only for copper service lines, and is set to missing otherwise. These difference-in-differences models are estimated using OLS regression.

REFERENCES

1. P. J. Hammer, The Flint Water Crisis, the Karegnondi Water Authority and strategic-structural racism. *Crit. Socio.* **45**, (2019).

2. S. Zahran, S. P. Mcelmurry, R. C. Sadler, Four phases of the Flint Water Crisis: Evidence from blood lead levels in children. *Environ. Res.* **157**, 160 –172 (2017).

3. M. Hanna-Attisha, J. LaChance, R. C. Sadler, A. C. Schnepp, Elevated blood lead levels in children associated with the flint drinking water crisis: A spatial analysis of risk and public health response, *Am. J. Public Health*, **106**, 283–290 (2016).

4. A. S. Winter, R. J. Sampson, From lead exposure in early childhood to adolescent health: A chicago birth cohort, *Am. J. Public Health* **107**, 1496–1501, (2017).

5. G. A. Washerman, B. Staghezza-Jaramillo, P. Shrout, D. Popovac, J. Graziano, The effect of lead exposure on behavior problems in preschool children. *Am. J. Public Health* **88**, 481 –486 (1998).

6. R. L. Canfield, M. H. Gendle, D. A. Cory-Slechta, Impaired neuropsychological functioning in lead-exposed children. *Dev. Neuropsychol.* **26**, 513 –540 (2004).

7. M. S. Amato, C. F. Moore, S. Magzamen, P. Imm, J. A. Havlena, H. A. Anderson, M. S. Kanarek, Lead exposure and educational proficiency: Moderate lead exposure and educational proficiency on end-of-grade examinations. *Ann. Epidemiol.* **22**, 738 –743 (2012).

8. K. M. Cecil, C. J. Brubaker, C. M. Adler, K. N. Dietrich, M. Altaye, J. C. Egelhoff, S. Wessel, I. Elangovan, R. Hornung, K. Jarvis, B. P. Lanphear, Decreased brain volume in adults with childhood lead exposure. *PLoS Med.* **5**, e112 (2008).

9. A. L. Beckley, A. Caspi, J. Broadbent, H. Harrington, R. M. Houts, R. Poulton, S. Ramrakha, A. Reuben, T. E. Moffitt, Association of childhood blood lead levels with criminal offending. *JAMA Pediatr.* **172**, 166 –173 (2018).

10. A. Reuben, A. Caspi, D. W. Belsky, J. Broadbent, H. Harrington, K. Sugden, R. M. Houts, S.

Ramrakha, R. Poulton, T. E. Moffitt, Association of childhood blood lead levels with cognitive function and socioeconomic status at age 38 years and with IQ change and socioeconomic mobility between childhood and adulthood. *JAMA* **317**, 1244 –1251 (2017).

11. S. K. Brooks, S. S. Patel, Psychological Consequences of the Flint Water Crisis: A Scoping Review. *Disaster Med. Public Health Prep.* **16**, 1259 –1269 (2022).

12. A. Abadie, Using synthetic controls: Feasibility, data requirements, and methodological aspects. *J. Econ. Lit.* **59**, 391–425 (2021).

13. E. Ben-Michael, A. Feller, J. Rothstein, The augmented synthetic control method. *J. Am. Stat. Assoc.* **116**, 1789 –1803 (2021).

14. D. Arkhangelsky, S. Athey, D. A. Hirshberg, G. W. Imbens, S. Wager, Synthetic difference-in-differences. *Am. Econ. Rev.* **111**, 4088 –4118 (2021).

15. S. Zahran, D. Mushinski, S. P. McElmurry, C. Keyes, Water lead exposure risk in Flint, Michigan after switchback in water source: Implications for lead service line replacement policy. *Environ. Res.* **181**, 108928 (2020).

16. A. Reuben, M. L. Elliott, W. C. Abraham, J. Broadbent, R. M. Houts, D. Ireland, A. R. Knodt, R. Poulton, S. Ramrakha, A. R. Hariri, A. Caspi, T. E. Moffitt, Association of childhood lead exposure with MRI measurements of structural brain integrity in midlife. *JAMA* **324**, 1970 –1979 (2020).

17. T. I. Lidsky, J. S. Schneider, Lead neurotoxicity in children: Basic mechanisms and clinical correlates. *Brain* **126**, 5 –19 (2003).

18. C. Muller, R. J. Sampson, A. S. Winter, Environmental inequality: The social causes and consequences of lead exposure. *Annu. Rev. Sociol.* **44**, 263 –282 (2018).

19. G. T. Wodtke, S. Ramaj, J. Schachner, Toxic neighborhoods: The effects of concentrated poverty and environmental lead contamination on early childhood development. *Demography,* **59**, 1275–1298 (2022).

20. S. B. Billings, K. T. Schnepel, Life after lead: Effects of early interventions for children exposed to lead. *Am. Econ. J. Appl. Econ.* **10**, 315 –344 (2018).

21. A. Aizer, J. Currie, P. Simon, P. Vivier, Do low levels of blood lead reduce children's future test scores? *Am. Econ. J. Appl. Econ.* **10**, 307 –341 (2018).

22. L. C. Sorensen, A. M. Fox, H. Jung, E. G. Martin, Lead exposure and academic achievement: Evidence from childhood lead poisoning prevention efforts. *J. Popul. Econ.* **32**, 179 –218 (2019).

23. H. Grönqvist, J. P. Nilsson, P. O. Robling, Understanding how low levels of early lead exposure affect children's life trajectories. *J. Polit. Econ.* **128**, 3376 –3433 (2020).

24. W. Troesken, Lead water pipes and infant mortality at the turn of the twentieth century. *J. Hum. Resour.* **43**, 553 –575 (2008).

25. J. P. Ferrie, K. Rolf, W. Troesken, "Cognitive disparities, lead plumbing, and water chemistry: Prior exposure to water-borne lead and intelligence test scores among World War Two U.S. Army enlistees," *Econ. Hum. Biol.* **10**, 98–111 (2012).

26. J. J. Feigenbaum, C. Muller, Lead exposure and violent crime in the early twentieth century. *Explor. Econ. Hist.* **62**, 51 –86 (2016).

27. R. W. Hornung, B. P. Lanphear, K. N. Dietrich, Age of greatest susceptibility to childhood lead exposure: A new statistical approach. *Environ. Health Perspect.* **117**, 1309 –1312 (2009).

28. W. Troesken, *The Great Lead Water Pipe Disaster* (MIT Press, 2006).

29. R. Renner, Exposure on tap: Drinking water as an overlooked source of lead. *Environ. Health Perspect.* **118**, A68 –A72 (2010).

30. Chernobyl Forum, "Chernobyl's Legacy: Health, Environmental and Socio-Economic Impacts and Recommendations to the Governments of Belarus, the Russian Federation and Ukraine," (International Atomic Energy Agency, 2006).

31. B. Pfefferbaum, S. J. Nixon, P. M. Tucker, R. D. Tivis, V. L. Moore, R. H. Gurwitch, R. S. Pynoos,

H. K. Geis, Posttraumatic stress responses in bereaved children after the Oklahoma City bombing. *J. Am. Acad. Child Adolesc. Psychiatry* **38**, 1372 –1379 (1999).

32. B. Pfefferbaum, T. W. Seale, N. B. Mcdonald, E. N. Brandt, S. M. Rainwater, B. T. Maynard, B. Meierhoefer, P. D. Miller, Posttraumatic stress two years after the oklahoma city bombing in youths geographically distant from the explosion. *Psychiatry* **63**, 358 –370 (2000).

33. Y. Neria, L. DiGrande, B. G. Adams, Posttraumatic Stress Disorder Following the September 11, 2001, terrorist Attacks: A review of the literature among highly exposed populations. *Am. Psychol.* **66**, 429 –446 (2011).

34. S. Galea, J. Ahern, H. Resnick, D. Kilpatrick, M. Bucuvalas, J. Gold, D. Vlahov, Psychological sequelae of the September 11 terrorist attacks in New York City. *N. Engl. J. Med.* **346**, 982 –987 (2002).

35. A. W. Spell, M. Lou Kelley, J. Wang, S. Self-Brown, K. L. Davidson, A. Pellegrin, J. L. Palcic, K. Meyer, V. Paasch, A. Baumeister, The moderating effects of maternal psychopathology on children's adjustment post-Hurricane Katrina. *J. Clin. Child Adolesc. Psychol.* **37**, 553 –563 (2008).

36. P. Sharkey, The acute effect of local homicides on children's cognitive performance. *Proc. Natl. Acad. Sci. U.S.A.* **107**, 11733 –11738 (2010).

37. M. Rossin-Slater, M. Schnell, H. Schwandt, S. Trejo, L. Uniat, Local exposure to school shootings and youth antidepressant use. *Proc. Natl. Acad. Sci. U.S.A.* **117**, 23484 –23489 (2020).

38. S. Gershenson, E. Tekin, The effect of community traumatic events on student achievement: Evidence from the beltway sniper attacks. *Educ. Financ. Policy* **13**, 513 –544 (2018).

39. C. Omoeva, W. Moussa, R. Hatch, F. Affolter, B. Jang, A. Azaryeva-Valente, D. Kelly, D. Richardson, H. Hattori, N. Balvin, The Effects of armed conflict on educational attainment and inequality, **18**, 1 (2018).

40. F. Torche, A. Villarreal, Prenatal exposure to violence and birth weight in mexico: selectivity, exposure, and behavioral responses. *Am. Sociol. Rev.* **79**, 966 –992 (2014).

41. S. Gershenson, M. S. Hayes, Police shootings, civic unrest and student achievement: Evidence from Ferguson. *J. Econ. Geogr.* **18**, 663 –685 (2018).

42. S. Rosenfield, Labeling mental illness: The effects of received services and perceived stigma on life satisfaction. *Am. Sociol. Rev.* **62**, 660 (1997).

43. D. Pager, The mark of a criminal record. *Am. J. Sociol.* **108**, 937 –975 (2003).

44. E. L. Green, "Flint's children suffer in class after years of drinking the lead-poisoned water," *The New York Times*, 6 November 2019; www.nytimes.com/2019/11/06/us/politics/flint-michigan-schools.html.

45. H. F. Gómez, K. Dietrich, "The children of flint were not 'poisoned," *The New York Times*, 22 July 2018; www.nytimes.com/2018/07/22/opinion/flint-lead-poisoning-water.html.

46. B. J. Goosby, J. E. Cheadle, C. Mitchell, Stress-related biosocial mechanisms of discrimination and African American Health Inequities. *Annu. Rev. Sociol.* **44**, 319 –340 (2018).

47. S. J. Spencer, C. Logel, P. G. Davies, Stereotype threat. *Annu. Rev. Psychol.* **2016**, 32 (2016).

48. T. Aquino, J. E. Brand, F. Torche, Unequal effects of disruptive events, *Sociol. Compass* **16**, 1–16 (2022).

49. F. Torche, Prenatal exposure to an acute stressor and children's cognitive outcomes. *Demography* **55**, 1611 –1639 (2018).

50. D. Almond, L. Edlund, M. Palme, Chernobyl's subclinical legacy: Prenatal exposure to radioactive fallout and school outcomes in Sweden. *Q. J. Econ.* **124**, 1729 –1772 (2009).

51. E. M. Fahle, T. J. Kane, T. Patterson, S. F. Reardon, "School District and Community Factors Associated With Learning Loss During the COVID-19 Pandemic" (2023); https://cepr.harvard.edu/sites/hwpi.harvard.edu/files/cepr/files/explaining_c.

52. S. J. Masten, S. H. Davies, S. P. McElmurry, Flint water crisis: What happened and why? *J. Am. Water Works Assoc.* **108**, 22 –34 (2016).

53. K. J. Pieper, M. Tang, M. A. Edwards, Flint Water Crisis caused by interrupted corrosion control: investigating ‘ground zero’ home. *Environ. Sci. Technol.* **51**, 2007 –2014 (2017).

54. P. Christensen, D. A. Keiser, G. E. Lade, Economic effects of environmental crises: Evidence from Flint, Michigan. *Am. Econ. J. Econ. Policy* **15**, 196–232 (2023).

55. M. A. S. Laidlaw, G. M. Filippelli, R. C. Sadler, C. R. Gonzales, A. S. Ball, H. W. Mielke, Children’s blood lead seasonality in flint, Michigan (USA), and soil-sourced lead hazard risks. *Int. J. Environ. Res. Public Health* **13**, 358 (2016).

56. D. S. Grossman, D. J. G. Slusky, The impact of the Flint Water Crisis on fertility. *Demography* **56**, 2005–2031 (2019).

57. R. Wang, X. Chen, X. Li, Something in the pipe: The Flint water crisis and health at birth. *J. Popul. Econ.* **35**, 1723–1749 (2022).

58. H. F. Gómez, D. A. Borgialli, M. Sharman, A. T. Weber, A. J. Scolpino, J. M. Oleske, J. D. Bogden, Blood lead levels in females of childbearing age in Flint, Michigan, and the water crisis. *Obstet. Gynecol.* **134**, 628 –635 (2019).

59. S. Danagoulian, D. Jenkins, Rolling back the gains: Maternal stress undermines pregnancy health after Flint’s water switch. *Heal. Econ. (United Kingdom)* **30**, 564 –584 (2021).

60. R. N. Jacobs, Civil society and crisis: Culture, discourse, and the Rodney King beating. *Am. J. Sociol.* **101**, 1238 –1272 (1996).

61. A. Krings, D. Kornberg, E. Lane, Organizing under austerity: How residents’ concerns became the Flint Water Crisis. *Crit. Socio.* **45**, 583 –597 (2019).

62. K. Michelmore, S. Dynarski, The Gap within the gap. *AERA Open* **3**, 1 –18 (2017).

63. J. Abernethy, A. Chojnacki, A. Farahi, E. Schwartz, J. Webb, Active remediation: The search for lead pipes in Flint, Michigan. *Proc. ACM SIGKDD Int. Conf. Knowl. Discov. Data Min.* **1**, 5 –14 (2018).

64. A. Abadie, J. Gardeazabal, The economic costs of conflict: A case study of the Basque country. *Am. Econ. Rev.* **93**, 113 –132 (2003).

65. A. Abadie, A. Diamond, J. Hainmueller, Synthetic control methods for comparative case studies: Estimating the effect of California's tobacco control program. *J. Am. Stat. Assoc.* **105**, 493 –505 (2010).

66. N. Doudchenko, G. W. Imbens, "Balancing, regression, difference-in-differences and synthetic control methods: A synthesis" (National Bureua of Economic Research, 2016).

67. A. Zeileis, Econometric computing with HC and HAC covariance matrix estimators, *J. Stat. Softw.* **11**, 1 (2004).

68. R. G. Miller, *The Jackknife—A Review* (Oxford Univ. Press, 1974), vol. 61, pp. 1–15.

69. M. A. Kraft, Interpreting effect sizes of education interventions. *Educ. Res.* **49**, 241 –253 (2020).

70. K. Alexander, S. Pitcock, M. C. Boulay, "*The Summer Slide: What We Know and Can Do about Summer Learning Loss*" (Teachers College Press, 2016), 342 pp.

71. S. Trejo, G. Yeomans-Maldonado, B. Jacob, The Psychosocial Effects of The Flint Water Crisis On School-Age Children (National Bureua of Economic Research, 2021; www.nber.org/papers/w16019.

72. J. Sauve-Syed, Three essays on public policy and health. Thesis, 2018, Syracuse University, Syracuse, New York (2018); http://ovidsp.ovid.com/ovidweb.cgi?T=JS&PAGE=reference&D=psyc14&NEWS=N&AN=2016-58394-093.

73. P. Muennig, The social costs of lead poisonings. *Health Aff.* **35**, 1545 (2016).

74. B. P. Lanphear, R. Hornung, J. Khoury, K. Yolton, P. Baghurst, D. C. Bellinger, R. L. Canfield, K. N. Dietrich, R. Bornschein, T. Greene, S. J. Rothenberg, H. L. Needleman, L. Schnaas, G. Wasserman, J. Graziano, R. Roberts, Low-level environmental lead exposure and children's intellectual function: An international pooled analysis. *Environ. Health Perspect.* **113**, 894 –899

(2005).

75. D. B. Rubin, Causal inference using potential Outcomes. *J. Am. Stat. Assoc.* **100**, 322 –331 (2005).

76. B. Ferman, C. Pinto, Inference in differences-in-differences with few treated groups and heteroskedasticity. *Rev. Econ. Stat.* **101**, 452 –467 (2019).

77. T. G. Conley, C. R. Taber, Inference with ‘difference in differences’ with a small number of policy changes. *Rev. Econ. Stat.* **93**, 113 –125 (2011).

78. C. de Chaisemartin, X. D’Haultfœuille, Two-Way Fixed Effects Estimators with Heterogeneous Treatment Effects. *Am. Econ. Rev.* **110**, 2964 –2996 (2020).

79. K. Borusyak, X. Jaravel, J. Spiess, Revisiting event study designs: Robust and efficienct estimation. *Work Prog.*, 1 –48 (2021).

80. B. Callaway, P. H. C. Sant’Anna, Difference-in-Differences with multiple time periods. *J. Econom.* **225**, 200 –230 (2021).
